# CT-Derived Radiomic Signature of MUC6 Expression Improves Guideline-Based Risk Stratification in Intraductal Papillary Mucinous Neoplasms

**DOI:** 10.3390/cancers18142264

**Published:** 2026-07-15

**Authors:** Evan W. Davis, Margaret A. Park, Toni L. Basinski, Solomon Alhassan, Maria F. Gomez, Maria Genilo-Delgado, Andrew J. Sinnamon, Pamela J. Hodul, Aleksandra Karolak, Zena Sayegh, Jonathan Nguyen, Brittany Rummens, Jiannong Li, Aakash Tripathi, Nathan H. Parker, Jose M. Pimiento, Ghulam Rasool, Alexandra F. Tassielli, Dung-Tsa Chen, Barbara A. Centeno, Kun Jiang, Daniel Jeong, Jennifer B. Permuth

**Affiliations:** 1 Department of Cancer Epidemiology, H. Lee Moffitt Cancer Center and Research Institute, Tampa, FL 33612, USA; 2 Department of Gastrointestinal Surgical Oncology and Gastroenterology, H. Lee Moffitt Cancer Center and Research Institute, Tampa, FL 33612, USAmaria.genilodelgado@moffitt.org (M.G.-D.); allie.tassielli@moffitt.org (A.F.T.); 3 Department of Biostatistics and Bioinformatics, H. Lee Moffitt Cancer Center and Research Institute, Tampa, FL 33612, USA; jiannong.li@moffitt.org (J.L.);; 4 Department of Machine Learning, H. Lee Moffitt Cancer Center and Research Institute, Tampa, FL 33612, USA; 5 Tissue Core, H. Lee Moffitt Cancer Center and Research Institute, Tampa, FL 33612, USA; 6 Advanced Analytical and Digital Laboratory, H. Lee Moffitt Cancer Center and Research Institute, Tampa, FL 33612, USA; 7 Department of Health Outcomes and Behavior, H. Lee Moffitt Cancer Center and Research Institute, Tampa, FL 33612, USA; nathan.parker@moffitt.org; 8 Department of Anatomic Pathology, H. Lee Moffitt Cancer Center and Research Institute, Tampa, FL 33612, USA; 9 Department of Diagnostic Imaging, H. Lee Moffitt Cancer Center and Research Institute, Tampa, FL 33612, USA

**Keywords:** pancreatic cystic lesions, pancreatic ductal adenocarcinoma, image-based biomarkers, surgical decision-making

## Abstract

Background and Aims: Accurate pre-operative identification of high-risk intraductal papillary mucinous neoplasms (IPMNs) remains a major clinical challenge, particularly for branch-duct (BD) lesions where guideline-based criteria incompletely capture biologic aggressiveness. We investigated whether tumoral mucin expression identifies high-risk IPMN pathology (i.e., high-grade dysplasia or invasive carcinoma) and whether computed tomography (CT)-derived radiomic features can serve as non-invasive biomarkers to enhance pre-operative risk assessment beyond international consensus guidelines (ICG) criteria. Methods: Multiplex immunofluorescence quantified MUC1, MUC2, MUC5AC, and MUC6 expression in tissue microarrays from 101 surgically resected IPMNs classified as low-risk (low-grade dysplasia) or high-risk (high-grade dysplasia or invasive carcinoma). Associations were evaluated using Wilcoxon rank-sum tests, and their discriminatory capability evaluated using receiver operating characteristic curves. For mucins predictive of high-risk pathology, a CT-based ‘radiomic’ signature was developed. Incremental value beyond ICG criteria was evaluated using discrimination metrics and decision curve analysis. Results: Reduced MUC6 expression was significantly associated with high-risk pathology (*p* = 0.001) and had the highest discriminatory performance (AUC = 0.72). A CT-derived radiomic signature predictive of low MUC6 expression achieved an AUC of 0.75 and, when integrated with ICG high-risk stigmata (HRS), demonstrated improved discrimination and favorable decision-curve characteristics compared with HRS alone, including among BD-IPMNs. Conclusions: Loss of tumoral MUC6 expression is associated with high-risk IPMN pathology and may be approximated using CT-derived radiomic features, supporting the feasibility of non-invasive molecular phenotyping. These findings suggest that integration of molecular and imaging biomarkers with guideline-based criteria may enhance pre-operative IPMN risk stratification; however, prospective external validation in broader surveillance populations and multi-institutional cohorts is warranted prior to clinical implementation.

## Simple Summary

Pancreatic ductal adenocarcinoma (PDAC) incidence continues to rise, highlighting the need to improve early detection and prevention strategies. Because at least one-third of PDACs arise from cystic precursors called intraductal papillary mucinous neoplasms (IPMNs), their accurate risk stratification represents an important opportunity for cancer interception. Yet, current guideline-based approaches remain imperfect and may result in overtreatment and undertreatment. Our study demonstrates that reduced tumoral MUC6 expression is associated with high-risk pathology and that a quantitative CT-derived radiomic signature can non-invasively capture this molecular phenotype. When integrated with established clinical and imaging features, MUC6 and its radiomic signature improved discrimination of high-risk pathology beyond guideline-based assessment, including among branch-duct IPMNs where management uncertainty is greatest. Pending external validation, these findings suggest that radiomic analysis of routinely acquired imaging may provide a non-invasive marker of IPMN biology and support more precise surgical decision-making.

## 1. Introduction

Pancreatic cancer is one of the most lethal malignancies in the United States, with a five-year relative survival of only 13.3% [1], and is projected to be the second leading cause of cancer deaths by 2030 [2]. These low survival rates can be partly attributed to most patients being diagnosed with advanced disease (3.2% five-year relative survival [1]), precluding them from surgical resection. However, even those diagnosed with localized disease who undergo surgical resection only have a five-year relative survival of 43.6% [1]. Even more concerning is the increasing incidence of pancreatic cancer [1], especially among younger individuals [3], demonstrating the need to improve early detection and prevention strategies.

Roughly one-third of pancreatic ductal adenocarcinomas (PDAC), the most common histologic subtype of pancreatic cancer, arise from cystic precursor lesions called intraductal papillary mucinous neoplasms (IPMNs) [4]. Accurate characterization of IPMNs offers an opportunity for early detection and interception of pancreatic lesions with a high risk of progression to malignancy. Yet, IPMNs are challenging to manage due to a lack of radiologic and molecular markers that can distinguish between IPMNs harboring low-grade dysplasia (LGD) that warrant active surveillance versus high-grade dysplasia (HGD) or invasive carcinoma that warrant surgical resection [5,6]. Currently, surgical resection is the only method to accurately determine IPMN severity, but surgical management of these neoplasms poses significant morbidity and mortality risks [7,8]. Thus, surgical resection is only ideal for patients with an IPMN suspected of harboring HGD or early invasion. To address this issue, international consensus guidelines (ICG) (also known as Kyoto Criteria) have been developed to predict the final pathology of IPMNs using conventional radiologic and clinical features [9,10]. Unfortunately, using ICG criteria for treatment stratification contributes to both undertreatment and overtreatment [11,12]. Therefore, relying solely on ICG to inform treatment decisions may be insufficient due to subtle molecular differences in IPMNs with similar radiologic and clinical presentations.

Mucin glycoprotein expression may provide important insight into these molecular differences. While expression patterns of MUC1, MUC2, MUC5AC, and MUC6 are used to classify the epithelial subtype of IPMNs [13], which can be informative for risk prediction and prognosis [14,15], evidence has emerged that tumoral expression of these mucins differs depending on severity of dysplasia or presence of invasion [16,17,18,19,20,21,22,23,24,25,26]. For example, MUC1 and MUC5AC expression have been shown to be higher in PDAC versus benign pancreatic conditions [16,18,20,22,25,26] and increase with increasing grade of dysplasia in precursor lesions [17,22,23]. Conversely, evidence suggests expression of both MUC2 and MUC6 decrease with increasing IPMN dysplasia and are lowest in PDAC [17,19,21,22,24,25,26]. MUC6 is of particular interest given its association with gastric-lineage differentiation, a phenotype generally linked to lower malignant potential, raising the possibility that loss of MUC6 reflects de-differentiation during neoplastic progression [27,28].

Evaluating tumoral mucin expression may provide clinically meaningful diagnostic utility for therapeutic risk stratification. However, prior studies have focused on distinguishing invasive carcinoma from benign conditions [29,30,31,32,33,34,35,36]. To our knowledge, no studies have systematically evaluated tumoral mucin expression for discriminating LGD from HGD or invasive IPMNs or PDAC using tissue specimens. The few studies that have explored mucin expression in low-versus and high-risk lesions are limited in sample size (*n* ≤ 86) and have relied on cyst fluid or extracellular vesicles in plasma rather than tumor tissue, which may more directly reflect underlying biology [37,38,39]. Moreover, only one such study incorporated imaging features included in ICG [39], and none have assessed non-invasive quantitative imaging approaches to approximate mucin expression despite growing evidence that radiomic features may serve as surrogate biomarkers of underlying IPMN biology [40,41,42]. Importantly, radiomics may capture lesion-wide phenotypic heterogeneity that cannot be fully appreciated through focal tissue sampling alone. Therefore, we aimed to: (1) evaluate tumoral mucin expression in surgically resected IPMNs as a predictor of IPMN pathology; (2) develop a CT-derived radiomic signature to pre-operatively approximate mucin expression patterns associated with high-risk IPMN pathology; and (3) assess the added value of mucin expression associated with high-risk pathology and their radiomic signatures with guideline-based assessment for pre-operative risk stratification. As an exploratory analysis, we aimed to assess the added value of relevant mucin expression patterns and their radiomic signatures with ICG criteria in patients with BD-IPMNs. We hypothesized that specific mucin expression patterns (i.e., high MUC1, low MUC2, high MUC5AC, or low MUC6) integrated with ICG would improve risk prediction, and that a corresponding radiomic signature could serve as a non-invasive surrogate for tumoral mucin expression when predicting final pathology.

## 2. Materials and Methods

### 2.1. Study Population

In this retrospective cohort study, participants were included if they (1) underwent surgical resection of a pancreatic mass at Moffitt Cancer Center (Tampa, FL, USA) between 2001 and 2016, (2) their tumors were pathologically confirmed as IPMN or a PDAC with an associated IPMN, (3) had tissue available for tissue microarray (TMA) construction, (4) available tissue was representative of final pathology, and (5) had complete data available for clinically relevant covariates including high-risk stigmata (HRS), worrisome features (WFs), and duct involvement (Figure S1). This study was approved by the Moffitt Cancer Center Scientific Review Committee (MCC 20105) and Advarra Institutional Review Board (20192596). All patients provided written informed consent for participation.

### 2.2. TMA Construction

Using surgically resected tumor tissue samples stored as formalin-fixed paraffin-embedded (FFPE) tissue blocks, TMAs were generated using standard procedures in the Tissue Core shared resource at Moffitt Cancer Center. Briefly, hematoxylin and eosin (H&E) stained sections were overlaid on the surface of the FPPE blocks and marked by a board-certified GI pathologist (KJ, BAC) to confirm diagnoses and identify grades of dysplasia according to World Health Organization (WHO) Guidelines [benign pancreas epithelium, LGD, moderate grade dysplasia (MGD), HGD, and invasive carcinoma] [43,44]. In accordance with updated WHO guidelines [43], patients who were initially identified as having MDG were reclassified as having LGD. To account for tumor heterogeneity, duplicate core punches measuring 1mm in diameter were taken from regions of interest (ROI) on the FFPE blocks corresponding to the highest grade of dysplasia noted in the surgical pathology report and precisely arrayed into a new paraffin block using Manual tissue arrayer Model MTA-1 (Beecher Instruments, Inc., Sun Prairie, WI, USA).

### 2.3. Multiplex Immunofluorescence (mIF)

The newly generated TMA block was sectioned into 4 μm slices, and each TMA core was individually reviewed as a quality control measure. Cores with folded tissue, irregular core punches, or extensive tissue damage during procurement were excluded from mIF analyses. After quality control, IPMN TMA sections were immunostained using the AKOYA Biosciences OPAL TM 7-Color Automation IHC kit (Akoya Biosciences, Waltham, MA, USA) on the BOND RX autostainer (Leica Biosystems, Vista, CA, USA) using standard operating procedures. Autofluorescence slides (negative control) were included, which used primary and secondary antibodies omitting the OPAL fluorophores and DAPI. All TMA slides were stained in a single batch to prevent batch effects. Antibodies for the mucins of interest included MUC1 [Cell Marque, MRQ-17 (RRID:AB_1160625), HIER-EDTA pH 9.0, 1:400, dye 570], MUC2 [Cell Marque, MRQ-18 (RRID:AB_1160642), HIER- EDTA pH 9.0, 1:800, dye520], MUC5AC [Cell Marque, MRQ-19 (RRID:AB_1160649), HIER-EDTA pH 9.0, 1:1200, dye 650], and MUC6 [Cell Marque, MRQ-20 (RRID:AB_1160667), HIER-EDTA pH 9.0, 1:600, dye620]. Additional details on the mIF and antibody validation are available in the Supplemental Materials. All slides were imaged with the AKOYA PhenoImager HT Imaging System (Akoya Biosciences, Waltham, MA, USA) in the Advanced Analytical and Digital Laboratory shared resource at Moffitt Cancer Center.

### 2.4. Quantitative Image Analysis

Multi-layer TIFF image files were exported from InForm (AKOYA) and loaded into HALO (Indica Labs, Albuquerque, NM, USA) for quantitative image analysis. The tissue was segmented into individual cells using the DAPI marker. Positivity thresholds were determined per marker based on published staining patterns and intensity for each antibody. After setting a positive fluorescent threshold for each marker, the entire image set was analyzed with the created algorithm. The output included positive cell counts for each marker, and the percentage of cells positive for each marker was calculated as the number of positive cells divided by the total number of cells in each core multiplied by 100. Mean percent positivity for each marker was then generated for each patient by averaging the percent positivity from duplicate cores of the same grade. Inter-core variability in mucin expression was evaluated using a two-way random effects intraclass correlation coefficient (ICC).

### 2.5. Pathologic Risk Classification

IPMNs with LGD were classified as “Low-Risk” and IPMNs with at least one focus of HGD or invasion were classified as “High-Risk”. Additionally, PDACs with an associated IPMN were classified as “High-Risk”. In subset analyses, we further divided the “High-Risk” group into “High-Risk IPMN” for individuals with IPMNs bearing at least one focus of HGD and “Invasive IPMN/PDAC” for individuals with at least one invasive focus or a PDAC with an associated IPMN.

### 2.6. International Consensus Guidelines

Key clinical and radiological data to evaluate the presence of HRS and WFs according to ICG [9] were abstracted from the electronic medical record when available. Patients are defined as having high-risk stigmata (HRS) present if obstructive jaundice, an enhancing mural nodule or solid component measuring ≥5 mm, and/or main pancreatic duct measuring ≥10 mm are observed radiologically. They are considered to have worrisome features (WFs) present if one of the following clinical or radiological features is documented: acute pancreatitis, elevated CA 19-9, new onset of diabetes, an enhancing mural nodule measuring <5 mm, thickened/enhancing cyst walls, main pancreatic duct dilation ≥5 but <10 mm, an abrupt change in duct caliber with distal pancreatic atrophy, and/or lymphadenopathy.

### 2.7. CT Image Acquisition and Radiomic Feature Extraction

Radiomics data were available for a subset of patients with TMA data (*n* = 82; Table S1); imaging acquisition and feature extraction methods have been previously described [41,42]. Briefly, pre-operative three-phase pancreas protocol CT images obtained within approximately six months before surgery were analyzed, with most scans acquired using SIEMENS Sensation 16 scanners (Table S1) by our lead diagnostic radiologist (DJ). General CT acquisition parameters are summarized in Table S2.

Using Healthmyne software version 13.3.13 [Genentech (RRID: SCR_003997), San Francisco, CA, USA], semi-automated radiologist-driven IPMN tumor segmentations were performed to generate lesion volumes of interest across each CT contrast phase series. Radiomic feature definitions adhered to Imaging Biomarker Standardization Initiative (IBSI) recommendations [45]. We extracted 306 Imaging Biomarker Standardization Initiative-based radiomic features including 48 first-order intensity, 39 textural, and 219 morphologic features from each CT contrast phase series for 918 total features [45]. Algorithms were developed by the Quantitative Imaging Core at Moffitt Cancer Center and used for feature extraction and quantification with custom workflows in the Definiens Platform. To reduce instability related to acquisition-dependent feature scaling, radiomic features were standardized prior to downstream analyses.

### 2.8. Statistical Analysis

All analyses were performed in R version 4.5.2 (RRID: SCR_00432). First, we tested for differences in clinical covariates according to risk classification using Fisher’s exact test for categorical variables and Wilcoxon rank-sum tests using the ‘rstatix’ package [46]. Differences in mucin expression according to relevant clinical and demographic covariates were tested with Wilcoxon rank-sum or Kruskal–Wallis tests for binary and three or more level covariates, respectively, with the ‘rstatix’ package [46].

In primary analyses, we first performed a Box–Cox transformation with a 0.01 constant using the ‘caret’ package [47] followed by a Z-score standardization of MUC1, MUC2, MUC5AC, MUC6, and serum CA-19.9 (positive control) before testing for differences according to binary risk classification using a Wilcoxon rank-sum test and three-level risk classification using a Kruskal–Wallis test with Benjamini–Hochberg adjustments for multiple comparisons using ‘rstatix’ [46]. Post hoc Dunn’s tests were performed following a significant result in the Kruskal–Wallis. Considering the use of mucin glycoprotein expression for subtyping IPMNs [13], we also tested for differences by risk classification in subgroups by epithelial subtype. In the event of a mixed epithelial subtype, we included patients in each subgroup if their tumor comprised the specified subtype.

Next, we assessed the discriminatory capability of these mucins and CA 19-9 using five-fold cross-validation stratified by grade of dysplasia, duct involvement, and ICG status. Within each training fold, we used 1000 stratified bootstrap resamples to generate areas under the curve (AUC), performance metrics, and optimal thresholds for each mucin to distinguish high-risk pathology based on Youden’s index. The presented AUCs, sensitivity, specificity, PPV, NPV, and selected thresholds represent the mean value and 95% confidence intervals (CIs) across cross-validation folds. Mucins with AUC ≥ 0.70 were dichotomized using the Youden’s index threshold and retained in subsequent analyses.

We then evaluated CT-based radiomic features as potential non-invasive biomarkers of relevant mucin expression profiles in a subset of patients with radiomic features data (*n* = 82; Figure S1). To reduce data dimensionality, we used Spearman correlation to identify collinear features (r ≥ 0.80) retaining features with the lower *p*-value per cluster. To minimize overfitting risk and identify radiomic features reproducibly associated with MUC6 expression, multiple resampling-based feature selection strategies were employed. Four feature selection methods were evaluated including (i) rank-sum, (ii) LIMMA, (iii) random forest, and (iv) LASSO using five-fold cross-validation stratified by mucin expression and risk classification repeated ten times with 100 bootstrap resamples and Z-score standardization per fold. Test-specific feature selection criteria included: (i) *p* < 0.05 across ≥50% of bootstraps per fold in ≥80% of repeats for rank-sum and LIMMA; (ii) median importance in the top 20th percentile across bootstraps in ≥80% of repeats for random forest; and (iii) ≥50% selection frequency across bootstraps in ≥80% of repeats for LASSO. The final feature set was Z-score standardized from the raw data prior to applying PCA for signature development with the first principal component (PC1) representing the radiomic signature of mucin expression. Wilcoxon rank-sum tests were used to test differences in the PC1 scores by relevant dichotomized mucin expression variables. ROC curves were then used to assess the discriminatory capability of the radiomic signature in discerning tumoral mucin expression levels. For radiomic signatures with an AUC ≥ 0.70, Youden’s index was used to determine the optimal PC1 score cutoff for discerning between low and high mucin expression.

To assess the value of including relevant mucin expression or their radiomic signatures with guideline-based criteria for pre-operative risk stratification, we used a data-driven penalized logistic regression to evaluate the association of HRS or WFs with and without relevant mucin expressions and their radiomic signatures with the ‘rms’ package [48]. Internal validation and model calibration were assessed using 1000 bootstrap resamples. Given the hierarchical nature of HRS and WFs for assessing IPMN severity pre-operatively [9], we tested the association of WFs with and without mucin expression in the subset of patients without HRS present (*n* = 56; Figure S1). Predicted probabilities of high-risk pathology were extracted from each model (i.e., HRS alone, HRS/mucin, HRS/mucin radiomics, WFs alone, WFs/mucin, and WFs/mucin radiomics) and their discriminatory ability was assessed using ROC curves in the ‘pROC’ package [49]. DeLong’s test was used to compare AUCs from the HRS/WFs alone against the models including mucin expression or radiomic signatures. Youden’s index thresholds were derived to dichotomize predicted probabilities to calculate sensitivity, specificity, PPV, and NPV with the ‘epiR’ package [50]. Integrated discrimination improvement (IDI) index was then calculated to assess the added value of mucin expression or the radiomic signatures with ICG [51]. Decision curve analysis (DCA) was also used to assess the clinical utility of including mucin expression or the radiomic signature with ICG when risk stratifying IPMNs using the ‘rmda’ package [52]. Additionally, we evaluated the relevance of these findings restricted to patients with branch-duct involvement (BD-IPMN) on radiologic imaging as an exploratory analysis, as these IPMNs can be more challenging to manage using ICG [53].

Finally, as an exploratory post hoc analysis, we evaluated the relationship between the MUC6-associated radiomic signature and a separate internally developed radiomic classifier of IPMN risk status within the same analytic subsets. The internally developed classifier was derived using the same radiomics cohort and imaging workflow but optimized independently for distinguishing low-versus high-risk pathology rather than predicting MUC6 expression. This exploratory analysis was performed to determine whether the MUC6-associated radiomic signature captured imaging characteristics distinct from broader radiomic risk classification patterns. Specifically, we assessed the degree of correlation between the signatures and evaluated whether the MUC6-associated radiomic signature remained associated with high-risk pathology after accounting for the separate radiomic classifier. First, we used Spearman correlation coefficient to determine the interrelationships between the two radiomic signatures. We then used penalized logistic regression to evaluate associations between the MUC6-associated radiomic signature and high-risk pathology in independent models, combined models, and models incorporating HRS or WFs. All tests were two-sided, *p* < 0.05 was used to define statistical significance, and a seed was set for all bootstrap resampling analyses to ensure reproducibility.

## 3. Results

### 3.1. Cohort Characteristics

The analytic cohort included tissue from 101 eligible patients for whom concordance exists between the highest grade of dysplasia (or invasion) represented on their surgical pathology report and the highest available grade of tissue represented in the TMA with complete clinically relevant covariate data (Figure S1). Of these, 25 (24.6%) were classified as low-risk and 76 (75.4%) as high-risk. Baseline demographic characteristics, history of pancreatitis or new-onset diabetes, and availability of radiomic data did not differ between groups (Table 1). As compared to low-risk lesions, high-risk lesions were more frequently associated with main duct involvement (67.1% vs. 12.0%), larger tumors (3.41 cm vs. 2.40 cm), main duct dilation (59.2% versus 12.0%), presence of one or more HRS (57.9% versus 4.0%) or WFs (92.1% versus 52.0%), and pancreatobiliary subtype (67.1% versus 40.0%). Additionally, individuals with high-risk pathology had higher CA-19.9 and bilirubin and lower albumin levels.

### 3.2. Mucin Expression Differs by Pathologic Risk

**Figure 1:**
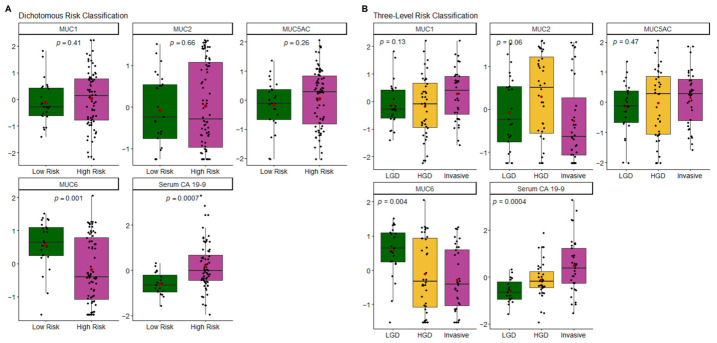
Boxplots showing Box–Cox-transformed and z-score-standardized mean percent positivity of mucin expression and serum CA 19-9 according to (**A**) dichotomous and (**B**) three-level pathologic risk classification including low-grade dysplasia (LGD), high-grade dysplasia (HGD), and invasive carcinoma.

When utilizing a dichotomous risk classification (Figure 1), patients with high-risk lesions had significantly lower MUC6 percent positivity (4.26% vs. 7.50%, *p* = 0.001) and significantly higher CA-19.9 compared with low-risk lesions (78.18 U/mL vs. 13.68 U/mL, *p* = 0.0007; Figure 1A and Table S3). When evaluated across three risk categories (low-risk, high-risk, invasive IPMN/PDAC), significant differences were once again observed for MUC6 (*p* = 0.004), and CA-19.9 (*p* = 0.0004; Figure 1B and Table S3). Post hoc testing revealed that differences in MUC6 positivity were pronounced between both low-risk and high-risk IPMN (7.50% vs. 5.53%, p_adj_ = 0.01) and low-risk IPMN vs. invasive disease (7.50% versus 2.84%, p_adj_ = 0.004; Tables S3 and S4). Differences in CA-19.9 were observed across all risk classification comparisons (Tables S3 and S4). Finally, trends in mucin expression differences by risk classification were largely consistent in subgroups by epithelial subtype (Figure S2).

### 3.3. Mucin Expression Is Associated with Clinical and Radiologic Characteristics

Differences in mucin expression and serum CA 19-9 according to clinicodemographic covariates are presented in Figure S3 and Table S5. No differences were observed according to age at diagnosis, BMI, history of pancreatitis, or duct involvement (Figure S3A,C,E,H). MUC1 was significantly higher in patients with new-onset diabetes (*p* = 0.009; Figure S3D) but no other differences were observed by new-onset diabetes. This finding is not unexpected given that diabetes is an established risk factor for pancreatic cancer. When examined according to HRS and WFs, as expected, CA19-9 was significantly higher in patients with at least one HRS or WF present (HRS *p* = 0.0009, Figure S3F; WFs *p* = 0.01, Figure S3G). Finally, MUC6 was significantly lower in patients with at least one HRS present (*p* = 0.04; Figure S3F) representing an intriguing finding related to existing guidelines for IPMN severity.

### 3.4. MUC6 Expression Is Associated with High-Risk IPMN Pathology

**Figure 2:**
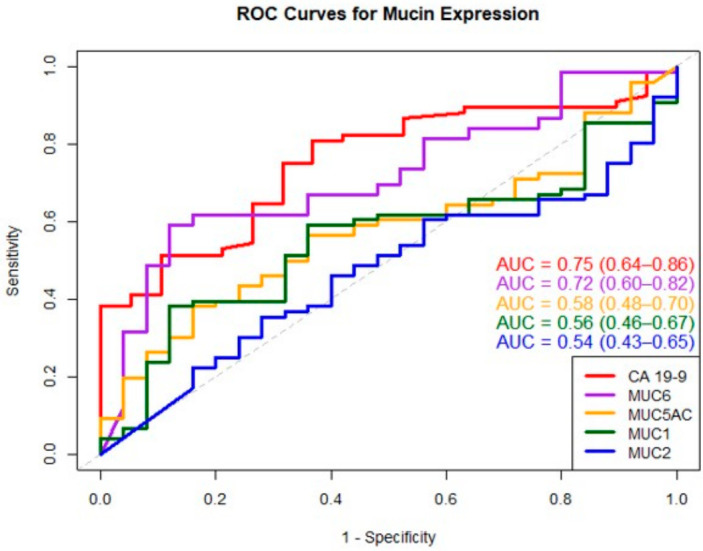
Receiver operating characteristic (ROC) curves with respective areas under the ROC curve (AUC) demonstrating the discriminatory capability of MUC1, MUC2, MUC5AC, MUC6, and serum CA 19-9 in distinguishing between low-risk and high-risk pathology.

Among evaluated mucins, MUC6 demonstrated the strongest discrimination between low- and high-risk pathology, yielding a mean AUC of 0.72 (95% CI: 0.60–0.82; Figure 2) and an optimal threshold of 1.53% positivity with 61% sensitivity, 84% specificity, 92% PPV, and 41% NPV (Table 2). This finding can be visually appreciated on representative low-risk and high-risk tissue cores with visibly greater MUC6 positivity in a patient with low-risk pathology than a patient with high-risk pathology (Figure S4). Conversely, MUC1, MUC2, and MUC5AC showed limited discriminatory performance with mean AUCs of 0.56, 0.54, and 0.58, respectively (Figure 2). Inter-core variability was minimal for MUC2 and MUC6 percent positivity (ICC = 0.91 and ICC = 0.75, respectively; Figure S5) while variability was substantial for MUC1 and MUC5AC (ICC = 0.56 and ICC = 0.59, respectively; Figure S5). Serum CA19-9 yielded a mean AUC of 0.75 (Figure 2); however, despite slightly higher specificity and PPV, CA19-9 demonstrated substantially lower sensitivity (43% versus 61%) and NPV (30% versus 41%) compared with MUC6 (Table 2).

### 3.5. A CT-Derived Radiomic Signature Correlates with Low MUC6 Expression

**Figure 3:**
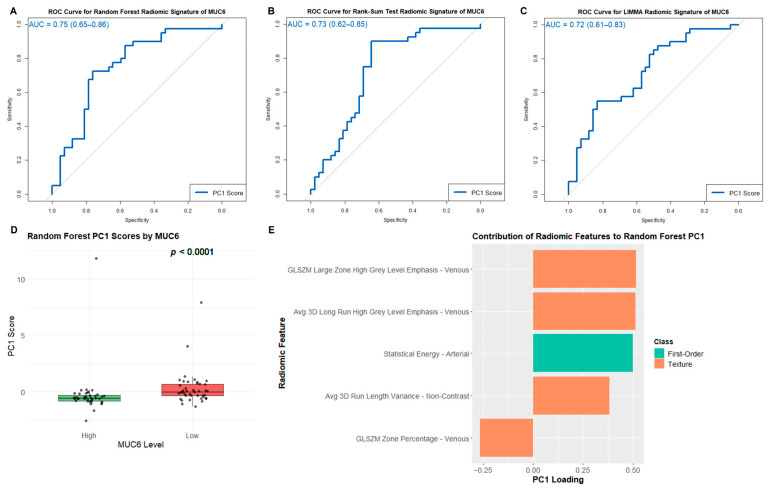
Results of the radiomic signature development for discriminating low MUC6. Receiver operating characteristic (ROC) curves and area under the ROC curve (AUC) are presented for the signatures developed using the (**A**) random forest, (**B**) rank-sum, and (**C**) LIMMA methods. The association of the random forest signature with risk classification is presented as a (**D**) boxplot with a Wilcoxon rank-sum comparison and (**E**) loading plot for the random forest signature. Abbreviations: SE = Sensitivity, SP = Specificity, PPV = Positive Predictive Value, NPV = Negative Predictive Value, PC1 = Principal Component 1, GLSZM = Grey Level Size Zone Matrix.

In the subset of patients with radiomics data (*n* = 82; Figure S1), PCA revealed no batch effects (Figure S4A) or effects by the year the CT scan was performed (Figure S4B). The random forest feature selection method yielded the best performance with moderate discrimination between low and high MUC6 expression (AUC = 0.75; Figure 3A–C) and PC1 scores significantly differed by MUC6 expression pattern (Figure 3D). The signature included five features (four textural and one first-order intensity) across all image phases with higher values of gray level size zone matrix (GLSZM) large zone high gray level emphasis, average 3D long run high gray level emphasis, and statistical energy contributing most to the radiomic signature (Figure 3E).

### 3.6. MUC6 Expression and Its Radiomic Signature Add Incremental Value to Consensus Guidelines

**Figure 4:**
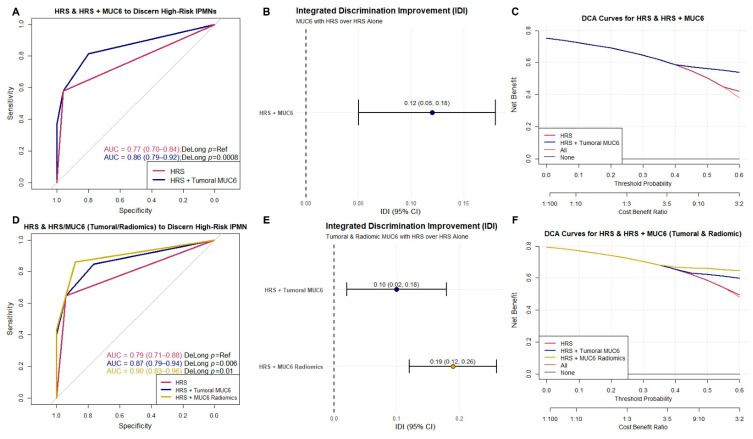
The utility of including tumoral MUC6 expression (**A**–**C**) and its radiomic signature (**D**–**F**) with guideline-based high-risk stigmata (HRS) for identifying patients with high-risk pathology. Receiver operatic characteristic (ROC) curves with area under the ROC curve (AUC) (**A**,**D**) depict the discrimination of HRS with and without MUC6 expression or its radiomic signature while forest plots (**B**,**E**) quantify the discrimination improvement for including MUC6 expression or its radiomic signature over HRS alone. Finally, decision curve analysis (DCA) curves (**C**,**F**) demonstrate the clinical utility of including MUC6 expression or its radiomic signature with HRS to aid in treatment decisions. Abbreviations: CI = Confidence Interval.

In the overall study population, including MUC6 with HRS had strong discrimination (AUC = 0.86; Figure 4A) superior to HRS alone (AUC = 0.77, DeLong *p* = 0.0008; Figure 4A) with stronger sensitivity (82% vs. 58%), NPV (59% vs. 43%), and accuracy (81.0% vs. 77.0%; Table 3). Internal validation revealed minimal optimism bias (optimism-corrected AUC = 0.77 and AUC = 0.85 for HRS alone and HRS/MUC6; Table S6) and strong model calibration (Brier scores of 0.15 and 0.12 for HRS alone and HRS/MUC6, respectively; Figure S7A,B). IDI and DCA revealed significant improvement including MUC6 (IDI = 0.10; Figure 4B) and net clinical benefit at ~40% threshold probability versus ~55% for HRS alone (Figure 4C). When the MUC6-associated radiomic signature was substituted for tumoral MUC6 expression, discrimination remained robust and significantly better than HRS alone (AUC = 0.90; DeLong *p* = 0.001; Figure 4D) with minimal optimism bias (Table S6) and strong model calibration (Figure S7E). IDI and DCA also revealed similar added value (IDI = 0.09; Figure 4E) and net benefit at lower threshold probability than HRS alone (Figure 4F). The slightly improved performance of the radiomic signature relative to tumoral MUC6 expression suggests that the imaging phenotype may capture additional lesion characteristics associated with high-risk pathology beyond MUC6 expression alone. Importantly, HRS with and without tumoral MUC6 expression performance was similar between the full cohort (*n* = 101; Figure S1) and radiomics subset (*n* = 82; Figure S1).

**Figure 5:**
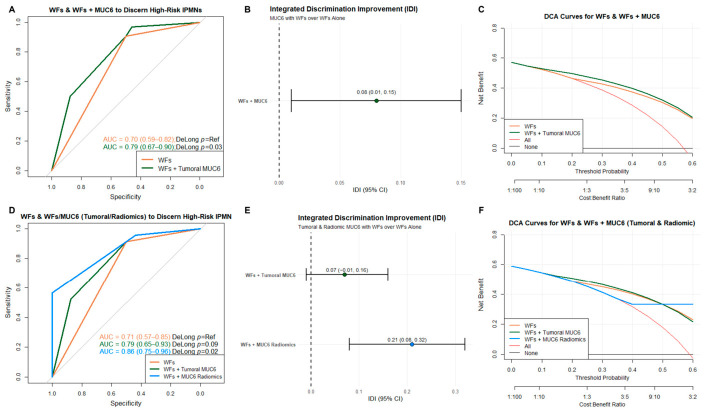
The utility of including tumoral MUC6 expression (**A**–**C**) and its radiomic signature (**D**–**F**) with guideline-based worrisome features (WFs) in patients without high-risk stigmata (HRS) present for identifying patients with high-risk pathology. Receiver operating characteristic (ROC) curves and area under the ROC curve (AUC) (**A**,**D**) depict the discrimination of WFs with and without MUC6 expression or its radiomic signature while forest plots (**B**,**E**) quantify the discrimination improvement for including MUC6 expression or its radiomic signature over WFs alone. Finally, decision curve analysis (DCA) curves (**C**,**F**) demonstrate the clinical utility of including MUC6 expression or its radiomic signature with WFs to aid in treatment decisions. Abbreviations: CI = Confidence Interval.

In the subset of patients without HRS present (*n* = 56; Figure S1), including MUC6 expression with WF status significantly improved discrimination compared with WFs alone (AUC = 0.79 vs. AUC = 0.70, DeLong *p* = 0.03; Figure 5A) with minimal optimism bias observed on internal validation (Table S6). Both models had moderate calibration (Figure S6A,B), although an S-shaped curve was observed for the WFs/MUC6 model, suggesting underprediction of high-risk lesions (Figure S6B). Despite only marginal differences in conventional performance metrics (Table 3), IDI and DCA analyses revealed incremental improvement over WFs alone (IDI = 0.08; Figure 5B), including net clinical benefit at lower threshold probabilities (~10% vs. ~20%; Figure 5C). When tumoral MUC6 expression was replaced with the MUC6 radiomic signature, discrimination further improved (AUC = 0.86; Figure 5D) with minimal optimism bias (Table S6) and moderate calibration, again with evidence of underprediction at higher prediction probabilities (Figure S8E). IDI analyses also revealed incremental value of the MUC6 radiomic signature beyond WFs alone (IDI = 0.17; Figure 5E). However, this improvement in discrimination was accompanied by substantial shifts in classification performance, including reduced sensitivity (57%) but 100% specificity (Table 3), resulting in limited incremental clinical utility of DCA relative to WFs alone or WFs combined with tumoral MUC6 expression (Figure 5F). Importantly, model performance of WFs with and without tumoral MUC6 expression was similar between the full non-HRS subset (*n* = 56) and the subset with radiomics data (*n* = 39; Figure S1).

### 3.7. MUC6 Expression May Be Particularly Useful in BD-IPMNs

**Figure 6:**
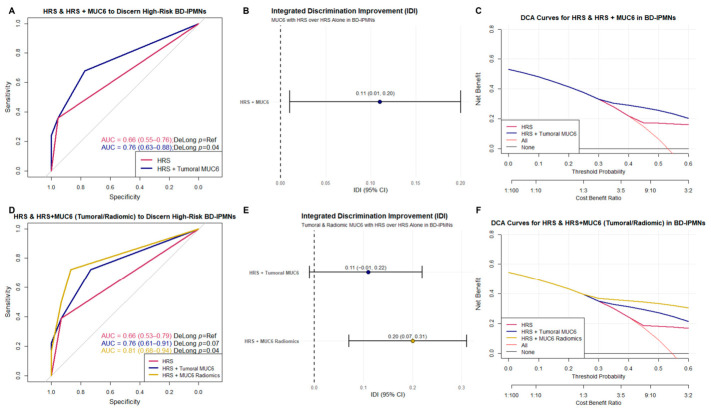
The utility of including tumoral MUC6 expression (**A**–**C**) and its radiomic signature (**D**–**F**) with guideline-based high-risk stigmata (HRS) for identifying BD-IPMN patients with high-risk pathology. Receiver operating characteristic (ROC) curves and area under the ROC curve (AUC) (**A**,**D**) depict the discrimination of HRS with and without MUC6 expression or its radiomic signature while forest plots (**B**,**E**) quantify the discrimination improvement for including MUC6 expression or its radiomic signature over HRS alone. Finally, decision curve analysis (DCA) curves (**C**,**F**) demonstrate the clinical utility of including MUC6 expression or its radiomic signature with HRS to aid in treatment decisions. Abbreviations: CI = Confidence Interval.

In the exploratory analyses restricted to patients with BD-IPMNs, including tumoral MUC6 expression with HRS yielded significantly better discrimination than relying on HRS alone (AUC = 0.76 vs. AUC = 0.66, DeLong *p* = 0.04; Figure 6A). Optimism bias was minimal (Table S7), and model calibration was moderate (Figure S9A,B). Improvements in sensitivity, NPV, and accuracy were observed over HRS alone (Table S8), and IDI (Figure 6B) and DCA (Figure 6C) revealed added value and clinical utility beyond HRS alone. When using the radiomic signature in lieu of tumor expression, model performance was largely similar to that observed in the full radiomics subset (Figure 6D–F and Figure S9C–E; Table S7). In the subset of BD-IPMN patients without HRS, findings were attenuated but not appreciably different than those in the full subset of patients without HRS with no clinical utility observed for including MUC6 nor its radiomic signature with WFs (Figures S10 and S11; Table S9).

### 3.8. Exploratory Comparison of the MUC6 Radiomic Signature with a Separate Risk Classification Signature

Exploratory post hoc analyses revealed moderate correlation between the MUC6-associated radiomic signature described in the current manuscript and a separate internally developed radiomic classifier of IPMN risk status evaluated within the same analytic subsets (Table S10). Despite partial overlap in radiomic features, the MUC6-associated signature remained consistently associated with high-risk pathology across multivariable models in the radiomics subsets (Table S11), suggesting that the signature may capture complementary imaging characteristics associated with biologically aggressive disease.

## 4. Discussion

Accurate pre-operative identification of high-risk IPMNs remains a clinical challenge, especially for BD-IPMNs, where current guideline-based criteria incompletely capture biologic aggressiveness. In this investigation, reduced MUC6 expression was consistently associated with high-risk pathology, and integration of MUC6 expression with ICG consistently improved discrimination, net clinical benefit, and NPV compared to ICG alone, including among patients with BD-IPMNs. Importantly, an independent CT-based radiomic signature was able to approximate this molecular phenotype non-invasively with similar performance when used as a surrogate biomarker and combined with HRS.

MUC6 expression was consistently lower in high-risk lesions and invasive disease. Notably, this difference in MUC6 expression was observed between patients with a low-risk versus high-risk IPMN and an invasive IPMN/PDAC suggesting that low tumoral MUC6 may be an important marker of high-risk pathology rather than just invasive carcinoma. These findings are largely concordant with the existing literature demonstrating reductions in MUC6 expression in PDAC tissue and with increasing dysplasia in precursor lesions, including IPMNs [19,21,22,24,25], suggesting loss of MUC6 as an aspect of lineage shift or de-differentiation accompanying malignant progression [54,55]. Our findings extend this body of work by demonstrating that tumoral MUC6 expression effectively discriminates low- from high-risk lesions, not merely invasive carcinoma from benign tissue. Additionally, MUC6 is characteristically expressed in gastric-type IPMNs and is associated with normal pyloric gland differentiation, whereas pancreatobiliary-type lesions, which are known to harbor greater malignant potential, often exhibit reduced MUC6 expression [24,27,54,55,56,57]. Moreover, the diffuse MUC6 positivity within the cytoplasm of cells on the apical luminal surface in a low-risk patient as compared to weak MUC6 positivity in a high-risk lesion (Figure S5) further supports the hypothesis that low MUC6 may be a marker of de-differentiation of normal pancreatic parenchyma to neoplastic epithelium or advanced histology, though mechanistic validation is required.

Importantly, MUC6 had the highest discriminatory power for distinguishing between IPMNs with low-risk and high-risk pathology outperforming standard clinical and radiologic criteria alone. The associated sensitivity and specificity of MUC6 were largely concordant with similar work by Yang et al. [39] although they found MUC5AC to have the highest discriminatory power. This difference may have arisen due to differences in biospecimen type (extracellular vesicles in plasma versus tumor tissue) considering that circulating mucin data can be influenced by non-cancerous conditions [58,59]. Nonetheless, our findings provide consistent evidence that tumoral MUC6 expression levels may improve pre-operative risk prediction for patients with IPMNs. Unlike CA 19-9, which may reflect broader pancreaticobiliary disease burden and systemic inflammation, MUC6 may more specifically capture lineage-associated IPMN biology and neoplastic progression. Including low tumoral MUC6 expression with HRS or WFs in patients without any HRS improved risk prediction, balancing sensitivity and specificity and yielding a greater net benefit than relying on ICG alone overall and among patients with BD-IPMNs which are more challenging to risk-stratify [53]. These findings further suggest that MUC6 may be particularly informative for reducing false reassurance in patients harboring high-risk lesions given its improved sensitivity and NPV relative to CA 19-9. Particularly notable is that combining tumoral MUC6 with WFs in the absence of HRS may be most useful in identifying individuals who harbor IPMNs that are truly low-risk as evidenced by the 92% and 91% NPV overall and among patients with BD-IPMNs, respectively. Although these findings are specific to tumoral MUC6 expression, a pre-operative radiomic signature of this phenotype may be a useful surrogate for direct measures.

We found that a signature of low MUC6 expression was able to approximate tumoral MUC6 expression and may serve as a surrogate, non-invasive biomarker of underlying IPMN biology distinct from, though partially overlapping with, a previously developed risk classification signature. Notably, the slightly improved discriminatory performance of the radiomic signature relative to tumoral MUC6 expression when combined with HRS/WFs suggests that the imaging phenotype may capture additional lesion characteristics associated with high-risk pathology beyond MUC6 expression alone. The identified radiomic features likely reflect imaging manifestations associated with altered mucin composition, epithelial density, and lesion architectural heterogeneity accompanying progression toward high-risk pathology. Partial overlap between the MUC6 radiomic signature and the previously developed high-risk/low-risk tumor radiomic signature, including shared contribution of statistical energy, further suggests that both signatures may capture complementary aspects of aggressive lesion architecture and biologic progression.

Features included in the MUC6 signature represent textural heterogeneity and lesion intensity distribution of CT voxels, with several features plausibly reflecting architectural alterations accompanying loss of MUC6 expression (i.e., reduced mucin content) and disease progression (i.e., increased epithelial density and/or solid components). Specifically, higher values of large zone high gray level emphasis, average 3D long run high gray level emphasis, statistical energy, and average 3D run length variance contributed to a higher PC1 score associated with low MUC6 expression, while lower values of zone percentage contributed to a higher PC1 score. Collectively, these findings suggest that tumors with low MUC6 expression exhibit reduced mucin-associated homogeneity and greater architectural complexity, supporting radiomics as a potential non-invasive marker of biologically aggressive IPMN phenotypes [60].

The greatest potential for downstream clinical integration lies in the comparable performance of the MUC6 radiomic signature to direct tissue measurement when incorporated into risk models with ICG. For instance, when the MUC6 radiomic signature was included with HRS, sensitivity, specificity, PPV, and NPV were all improved. Further, using the radiomic signature with HRS in patients with BD-IPMNs yielded similar results as when using tumoral MUC6 expression—a clinically relevant population in which management decisions often remain uncertain despite current guideline frameworks [9]. To this end, leveraging standard-of-care CT images pre-operatively to integrate a MUC6-based radiomic classifier could ultimately support multidisciplinary decision-making regarding surgical referral, surveillance intensity, or ancillary evaluation.

However, this observation was not consistent when the MUC6 signature was combined with WFs. This could be due, in part, to the limited sample size in this analytic subset, but could also be due to the MUC6 radiomic signature capturing information that, when combined with WFs, is more informative for ruling-out high-risk pathology than ruling-in given the weak sensitivity (57%) but strong specificity (100%). This pattern suggests that the MUC6 radiomic signature may preferentially identify a subset of lesions with particularly high biologic aggressiveness, albeit at the expense of sensitivity. Nevertheless, these findings should be interpreted with caution as prospective, external validation is necessary. In the meantime, alternative methods for assessing MUC6 expression pre-operatively should be explored given the observed utility of tumoral MUC6 expression combined with ICG for predicting high-risk pathology.

Fortunately, endoscopic ultrasound-guided fine-needle aspiration (EUS/FNA) procedures are commonplace diagnostic tools [61,62,63] that can be used to obtain cyst fluid and/or tissue samples to assess mucin expression pre-operatively [64,65], and have been shown yield similar mucin expression patterns as those evaluated using surgically resected tissue [66,67]. With the most recent update to ICG recommending cytological assessment when assessing for HRS [9], EUS/FNA procedures may be a feasible opportunity to pre-operatively evaluate MUC6 expression patterns during routine diagnostic workup. Given the added value and clinical utility of including tumoral MUC6 expression with WFs, including in BD-IPMNs, and the known difficulty in risk stratifying these patients, studies evaluating tissue samples obtained via biopsy for MUC6 expression are warranted. Regardless, large prospective studies evaluating radiomic features associated with mucin expression should we pursued to non-invasively assess underlying IPMN biology given the potential risks of EUS/FNA procedures [68,69,70]. Future investigations comparing tissue-based MUC6 expression with cyst fluid biomarkers and circulating molecular assays may further clarify the optimal strategy for biologically informed pre-operative IPMN risk stratification.

Several limitations should be considered when interpreting these findings. First, this retrospective, single-institution study includes only surgically resected cases, introducing spectrum bias and limiting generalizability to surveillance populations. The high prevalence of surgically resected high-risk lesions may also influence predictive performance metrics, particularly PPV and NPV. However, NPV remained superior with including MUC6 over ICG alone even at 20% outcome prevalence mitigating some concerns about spectrum bias. Nonetheless, prospective validation in a surveillance population is necessary. Second, the sample size is limited, especially when performing the radiomic feature analysis and BD-IPMN exploratory analyses. Even though model calibration ranges from moderate to excellent with Brier scores ranging from 0.09 to 0.21 (Figures S7–S9 and S11) and optimism-corrected discrimination (Tables S6 and S7) demonstrates that our findings are robust to internal validation, it is critical that the findings of this work, especially the cutoffs for low tumoral MUC6 expression and radiomic signature, be validated in larger data sets. Additionally, comparisons involving a separate internally developed radiomic classifier should be considered exploratory, pending independent validation and formal publication. Prospective assessment of model calibration across independent cohorts will also be essential prior to clinical translation. Third, radiomic features can be influenced by acquisition parameters and segmentation variability; external validation with harmonized imaging protocols, reproducibility testing, and calibration assessment is essential prior to clinical deployment. Future work incorporating MRI-based radiomics and multimodal imaging approaches may further improve biological characterization and predictive performance. Additionally, inter-observer reproducibility of lesion segmentation was not formally evaluated and remains an important consideration for future translational development and multi-institutional implementation. Fourth, TMA-based sampling may not fully capture spatial heterogeneity of mucin expression especially given that TMAs were generated from clinical collections potentially lending to the most concerning regions of tumor (i.e., regions with high MUC1 expression) being used for pathological analysis and thus unavailable for research purposes. However, since whole-lesion radiomics interrogates the entire tumor volume, it may complement tissue sampling and mitigate concerns related to intralesional heterogeneity. Additionally, there was minimal variability in MUC6 positivity between duplicate cores in our data, further mitigating this concern. Finally, while MUC6 demonstrated the strongest performance, additional mucins that were not evaluated in our investigation (e.g., MUC4, MUC16) [71] and other classes of biomarkers warrant investigation in future integrative models. Despite these limitations, this study represents one of the largest tissue-based evaluations of mucin expression in IPMNs and the first to link mucin biology with a CT-derived radiomic signature for pre-operative risk stratification.

## 5. Conclusions

Our findings suggest that relying on guideline-based clinical and radiologic criteria alone fails to capture molecular markers of IPMN aggression. Reduced tumoral MUC6 expression was associated with high-risk lesions, and this molecular phenotype can be approximated non-invasively using CT radiomics. Integration of radiomic–molecular biomarkers with established HRS improves pre-operative risk stratification, including in BD-IPMNs where ambiguity is highest, and may ultimately improve identification of patients most likely to benefit from resection while reducing unnecessary operative intervention. Indeed, the findings from this work are promising for refining pre-operative risk prediction but are largely hypothesis-generating and require critical steps be taken to advance this work. Key next steps to develop this work beyond a hypothesis include multi-institutional external validation with protocol harmonization, molecular validation, calibration assessment, and reproducibility testing, alongside evaluation in surveillance-intent cohorts to quantify impact on downstream management. Once validated, this approach could support the emerging paradigm of integrating molecularly informed imaging biomarkers with morphology-based guideline frameworks with the potential to reduce unnecessary harm and increase early detection and intervention of a highly lethal disease.

## Figures and Tables

**Table 1:** Clinical and epidemiological characteristics of the study population according to pathologic risk classification (*n* = 101).

Clinical & Epidemiological Variables	Low Risk *n* = 25 (24.6%)	High Risk *n* = 76 (75.4%)	*p*-Value
Age at Diagnosis (Years)	69.1 (±10.5)	71.0 (±10.0)	0.50
Sex			
Female	14 (56.0%)	33 (43.4%)	0.36
Male	11 (44.0%)	43 (56.6%)	
Race & Ethnicity			
Non-Hispanic White	22 (88.0%)	67 (88.2%)	1.00
Non-Hispanic Black	0 (0.0%)	3 (4.0%)	
Hispanic/Latinx	1 (4.0%)	3 (4.0%)	
Unknown ^a^	2 (8.0%)	3 (4.0%)	
Smoking Status			
Ever	11 (44.0%)	40 (52.6%)	0.49
Never	14 (56.0%)	35 (46.1%)	
Unknown ^a^	0 (0.0%)	1 (1.3%)	
Alcohol Consumption			
None/Mild	23 (92.0%)	63 (82.9%)	0.51
Moderate/Heavy	2 (8.0%)	12 (15.8%)	
Unknown ^a^	0 (0.0%)	1 (1.3%)	
Body Mass Index (kg/m^2^)	28.3 (±5.01)	26.5 (±4.19)	0.15
Body Mass Index Categories			
Underweight (BMI < 18.5)	0 (0.0%)	1 (1.3%)	0.73
Normal Weight (BMI 18.5–24.9)	7 (28.0%)	26 (34.2%)	
Overweight (BMI 25.0–29.9)	10 (40.0%)	32 (42.1%)	
Obese (BMI ≥ 30.0)	8 (32.0%)	17 (22.4%)	
New-Onset Diabetes Status			
Yes	2 (8.0%)	11 (14.5%)	0.51
No	23 (92.0%)	65 (85.5%)	
Pancreatitis History			
Acute Pancreatitis	1 (4.0%)	5 (6.6%)	0.56
Chronic Pancreatitis	5 (20.0%)	18 (23.7%)	
Acute & Chronic Pancreatitis	0 (0.0%)	1 (1.3%)	
Pancreatitis NOS	1 (4.0%)	11 (14.5%)	
None	18 (72.0%)	41 (54.0%)	
Ductal Communication			
Main Duct	3 (12.0%)	51 (67.1%)	**<0.0001**
Branch Duct	22 (88.0%)	25 (32.9%)	
Tumor Size (cm)	2.40 (±1.00)	3.41 (±1.76)	**0.007**
Tumor Size			
<3 cm	16 (64.0%)	32 (42.1%)	0.05
≥3 cm	7 (28.0%)	41 (54.0%)	
Unknown ^a^	2 (8.0%)	3 (4.0%)	
Main Duct Diameter (mm)	2.53 (±1.69)	6.93 (±5.12)	**<0.0001**
Main Duct Dilation			
None	21 (84.0%)	27 (35.5%)	**<0.0001**
Dilated 5–9 mm	3 (12.0%)	24 (31.6%)	
Dilated ≥ 10 mm	0 (0.0%)	21 (27.6%)	
Unknown ^a^	1 (4.0%)	4 (5.3%)	
High-Risk Stigmata			
Present (≥1)	1 (4.0%)	44 (57.9%)	**<0.0001**
Absent	24 (96.0%)	32 (42.1%)	
Worrisome Features			
Present (≥1)	13 (52.0%)	70 (92.1%)	**<0.0001**
Absent	12 (48.0%)	6 (7.9%)	
Epithelial Subtype			
Gastric	8 (32.0%)	2 (2.6%)	**0.003**
Intestinal	4 (16.0%)	18 (23.7%)	
Pancreatobiliary	9 (36.0%)	34 (44.7%)	
Gastric & Intestinal	2 (8.0%)	5 (6.6%)	
Gastric & Pancreatobiliary	0 (0.0%)	9 (11.8%)	
Intestinal & Pancreatobiliary	1 (4.0%)	5 (6.6%)	
Gastric, Intestinal, & Pancreatobiliary	0 (0.0%)	3 (4.0%)	
Unknown ^a^	1 (4.0%)	0 (0.0%)	
CA 19-9 Levels (continuous)	13.7 (±13.8)	469.5 (±1917.7)	**0.0007**
CA 19-9 Levels (categorical)			
Normal (<37)	17 (68.0%)	39 (51.3%)	**0.01**
High (≥37)	2 (8.0%)	29 (38.2%)	
Unknown ^a^	6 (24.0%)	8 (10.5%)	
Albumin Levels (continuous)	4.22 (±0.34)	3.90 (±0.64)	**0.01**
Albumin Levels (categorical)			
Normal (≥3.5)	24 (96.0%)	63 (82.9%)	0.18
Low (<3.5)	1 (4.0%)	13 (17.1%)	
Bilirubin Levels (continuous)	0.47 (±0.22)	0.88 (±1.28)	**0.02**
Bilirubin Levels (categorical)			
Normal (<0.3)	25 (100.0%)	64 (84.2%)	**0.04**
High (≥0.3)	0 (0.0%)	12 (15.8%)	
Radiomics Data			
Available	17 (68.0%)	65 (85.5%)	0.08
Unavailable	8 (32.0%)	11 (14.5%)	

*p*-Values < 0.05 are in bold font; differences in continuous variables were tested using Wilcoxon rank-sum tests; differences in categorical variables were tested using Fisher’s exact test; ^a^—Unknown groups were excluded from difference testing.

**Table 2:** Performance metrics and optimal thresholds for mucin expression and serum CA 19-9 in distinguishing low- from high-risk pathology (*n* = 101).

Biomarker	Sensitivity	Specificity	PPV	NPV	Optimal Threshold
MUC1	0.47 (0.36–0.59)	0.68 (0.46–0.85)	0.82 (0.67–0.92)	0.30 (0.18–0.43)	26.50 (11.41–33.99)
MUC2	0.34 (0.24–0.46)	0.76 (0.55–0.91)	0.81 (0.64–0.93)	0.28 (0.17–0.40)	1.72 (0.02–8.69)
MUC5AC	0.43 (0.32–0.55)	0.72 (0.51–0.88)	0.82 (0.67–0.93)	0.30 (0.19–0.43)	27.05 (6.58–45.79)
MUC6	0.61 (0.49–0.72)	0.84 (0.64–0.95)	0.92 (0.81–0.98)	0.41 (0.28–0.56)	1.53 (0.65–3.74)
CA 19-9 ^a^	0.43 (0.31–0.55)	0.89 (0.67–0.99)	0.94 (0.79–0.99)	0.30 (0.19–0.44)	-

Abbreviations: PPV = Positive Predictive Value; NPV = Negative Predictive Value; ^a^—Serum CA 19-9 was only available for 87 patients in the training cohort (19 Low Risk & 68 High Risk).

**Table 3:** Performance of HRS and WFs with and without MUC6 expression to discern high-risk IPMNs overall and in the subset of patients with radiomics data.

	Cohort Outcome Prevalence				20% Outcome Prevalence		
Risk Model	Sensitivity	Specificity	PPV	NPV	PPV	NPV	Accuracy ^a^
Overall (*n* = 101)							
HRS	0.58 (0.46–0.69)	0.96 (0.80–1.00)	0.98 (0.88–1.00)	0.43 (0.30–0.57)	0.68 (0.40–0.94)	0.90 (0.87–0.92)	77.0%
HRS + MUC6	0.82 (0.71–0.90)	0.80 (0.59–0.93)	0.93 (0.83–0.98)	0.59 (0.41–0.75)	0.49 (0.34–0.70)	0.94 (0.91–0.97)	81.0%
Patients with Radiomic Features Available (*n* = 82)							
HRS	0.65 (0.52–0.76)	0.94 (0.71–1.00)	0.98 (0.88–1.00)	0.41 (0.26–0.58)	0.63 (0.36–0.93)	0.91 (0.88–0.94)	79.5%
HRS + MUC6	0.85 (0.74–0.92)	0.76 (0.50–0.93)	0.93 (0.84–0.98)	0.57 (0.34–0.77)	0.46 (0.30–0.69)	0.95 (0.91–0.97)	80.5%
HRS + MUC6 Radiomics	0.86 (0.75–0.93)	0.88 (0.64–0.99)	0.97 (0.88–1.00)	0.62 (0.41–0.81)	0.59 (0.38–0.86)	0.96 (0.93–0.98)	87.0%
Patients without HRS Present (*n* = 56)							
WFs	0.91 (0.75–0.98)	0.50 (0.29–0.71)	0.71 (0.54–0.84)	0.80 (0.52–0.96)	0.31 (0.24–0.42)	0.95 (0.88–0.99)	70.5%
WFs + MUC6	0.97 (0.84–1.00)	0.46 (0.26–0.67)	0.70 (0.55–0.83)	0.92 (0.62–1.00)	0.31 (0.24–0.41)	0.97 (0.92–1.00)	71.5%
Patients without HRS with Radiomic Features Available (*n* = 39)							
WFs	0.91 (0.72–0.99)	0.50 (0.25–0.75)	0.72 (0.53–0.87)	0.80 (0.44–0.97)	0.64 (0.36–0.92)	0.91 (0.88–0.94)	70.5%
WFs + MUC6	0.96 (0.78–1.00)	0.44 (0.20–0.70)	0.71 (0.52–0.86)	0.88 (0.47–1.00)	0.46 (0.29–0.69)	0.95 (0.91–0.97)	70.0%
WFs + MUC6 Radiomics	0.57 (0.34–0.77)	1.00 (0.79–1.00)	1.00 (0.75–1.00)	0.62 (0.41–0.80)	0.60 (0.36–0.86)	0.96 (0.93–0.98)	78.5%

Abbreviations: HRS = High-Risk Stigmata, WFs = Worrisome Features, PPV = Positive Predictive Value, NPV = Negative Predictive Value. ^a^—Accuracy represents the mean of the sensitivity and specificity.

## Data Availability

The data supporting the conclusions of this study are available from the corresponding author upon reasonable request and subject to institutional review and data-sharing agreements.

## Supplementary Materials

The following supporting information can be downloaded at https://www.mdpi.com/article/10.3390/cancers18142264/s1. Figure S1: Flow chart depicting the inclusion and exclusion criteria leading to our final sample size; Figure S2: Boxplots showing Box–Cox-transformed and z-score-standardized mean percent positivity of mucin expression and serum CA 19-9 according to dichotomous (A–C) and three-level pathologic risk classification (D–F) in subgroups of patients with a gastric (A,D), intestinal (B,E), or pancreatobiliary (C,F) subtype; Figure S3: Boxplots showing mean percent positivity of mucin and other biomarker expression in pancreatic tumors according to several clinicodemographic factors including (A) age at diagnosis, (B) sex, (C) body mass index, (D) new-onset diabetes, (E) history of pancreatitis, (F) high-risk stigmata, (G) worrisome features, and (H) duct involvement; Figure S4: Tissue core images reflective of our central finding of higher MUC6 expression in patients with (A) low-risk lesions and lower expression in patients with (B) high-risk lesions using the 1.53% MUC6 positivity threshold; Figure S5: Variability in mucin expression per tissue core is presented as a forest plot of the intraclass correlation coefficient and their 95% confidence intervals (CIs). Variability between duplicate cores is minimal for MUC2 and MUC6, but high for MUC1 and MUC5AC; Figure S6: Analysis of batch effects by (A) analytic batch and (B) year of computed tomography (CT) scan effects across all radiomic features. Principal components analysis (PCA) scatterplot reveals no clustering by radiomics batch nor by year the CT scan was performed; Figure S7: Calibration plots and Brier scores of penalized logistic regression models of (A) HRS alone and (B) HRS with tumoral MUC6 expression in the overall study population along with (C) HRS alone, (D) HRS with tumoral MUC6 expression, and (E) HRS with the MUC6 radiomic signature in the subset with radiomics data; Figure S8: Calibration plots and Brier scores of penalized logistic regression models of (A) WFs alone and (B) WFs with tumoral MUC6 expression in the subset of patients without HRS present along with (C) WFs alone, (D) WFs with tumoral MUC6 expression, and (E) WFs with the MUC6 radiomic signature in the subset of patients without HRS present and radiomics data; Figure S9: Calibration plots and Brier scores of penalized logistic regression models of (A) HRS alone and (B) HRS with tumoral MUC6 expression in patients with BD-IPMNs along with (C) HRS alone, (D) HRS with tumoral MUC6 expression, and (E) HRS with the MUC6 radiomic signature in the BD-IPMN subset with radiomics data; Figure S10: The utility of including tumoral MUC6 expression (A–C) and its radiomic signature (D–F) with guideline-based WFs in patients without HRS present for identifying BD-IPMN patients with high-risk pathology. ROC curves (A,D) depict the discrimination of WFs with and without MUC6 expression or its radiomic signature while forest plots (B,E) quantify the discrimination improvement for including MUC6 expression or its radiomic signature over WFs alone. Finally, DCA curves (C,F) demonstrate the clinical utility of including MUC6 expression or its radiomic signature with WFs to aid in treatment decisions; Figure S11: Calibration plots and Brier scores of penalized logistic regression models of (A) WFs alone and (B) WFs with tumoral MUC6 expression in the subset of BD-IPMN patients without HRS present along with (C) WFs alone, (D) WFs with tumoral MUC6 expression, and (E) WFs with the MUC6 radiomic signature in the subset of BD-IPMN patients without HRS present and radiomics data; Table S1: CT scanners used to generate radiomic features data; Table S2: General triple phase contrast CT protocol used for assessment of IPMN patients; Table S3: Mean percent positivity of mucin expression and mean serum CA 19-9 levels according to pathologic risk classification; Table S4: Post hoc Dunn’s test for differences in mucin expression and serum CA 19-9 between individual levels of the three-tiered risk classification; Table S5: Mean percent positivity of mucin expression and mean serum CA 19-9 levels according to clinicodemographic covariates; Table S6: Optimism-corrected AUCs for HRS or WFs in patients without HRS with and without MUC6 expression or its radiomic signature; Table S7: Optimism-corrected AUCs for HRS or WFs in patients without HRS with and without MUC6 expression or its radiomic signature in patients with BD-IPMNs; Table S8: Performance of HRS with and without tumoral MUC6 expression and its radiomic signature to discern high-risk IPMNs overall and in BD-IPMN patients; Table S9: Performance of WFs in the absence of HRS with and without tumoral MUC6 expression and its radiomic signature to discern high-risk IPMNs overall and in BD-IPMN patients; Table S10: Spearman correlation coefficients of the MUC6 and risk classification radiomic signatures; Table S11: Odds ratios and 95% confidence intervals for the associations of HRS, WFs in patients without HRS, a previously developed tumor risk classification radiomic signature, and the MUC6 radiomic signatures assessing for independence of risk classification and MUC6 signatures when combined with HRS or WFs in patients without HRS; Supplemental Methods.
