# Supplementary material for: CT-Derived Radiomic Signature of MUC6 Expression Improves Guideline-Based Risk Stratification in Intraductal Papillary Mucinous Neoplasms
Source: Cancers (Basel). 2026 Jul 15;18(14):2264. doi: 10.3390/cancers18142264 (PMC13406335; doi:10.3390/cancers18142264)
Supplement: Supplementary file 1 [file cancers-18-02264-s001.zip › Supplementary Materials/Supplemental Tables.pdf]

Table S1. CT scanners used to generate radiomic features data.		
**Manufacturer**	**Model**	**Count**
SIEMENS	Biograph 6	1
	Sensation 16	36
	Sensation 40	14
	Sensation 64	19
	SOMATOM Definition AS	9
GE Medical Systems	LightSpeed16	1
	LightSpeed Pro 32	1
	Discovery CT750 HD	1

Table S2. General triple phase contrast CT protocol used for assessment of IPMN patients.			
**Acquisition Parameter**	**Non-Contrast**	**Arterial Phase**	**Venous Phase**
kVp	120	120	120
mA	Care Dose	Care Dose	Care Dose
Quality Reference mAs	150	150	150
Slice thickness/increment (mm)	3 x 3	3 x 3	3 x 3
Direction	Craniocaudal	Craniocaudal	Craniocaudal
Breath Holding	Held Inspiration	Held Inspiration	Held Inspiration
Scan Delay	None	ROI triggered: abdominal aorta near celiac axis (120 HU)	60 seconds post injection
Kernal	I30F	I30F	I30F

**Table S3. Mean percent positivity of mucin expression and mean serum CA 19-9 levels according to pathologic risk classification.**				
**Marker**	**Low Risk (N=25)**	**High Risk Combined (N=76)**	**High Risk IPMN (N=40)**	**Invasive IPMN/PDAC (N=36)**
MUC1	21.83 (±15.49)	26.38 (±19.39)	23.10 (±19.55)	30.01 (±18.81)
MUC2	3.26 (±9.60)	9.97 (±20.43)	12.66 (±23.37)	6.97 (±16.38)
MUC5AC	19.62 (±14.84)	22.67 (±4.26)	26.19 (±24.07)	27.36 (±21.32)
MUC6	7.50 (±6.56)	4.26 (±8.07)	5.53 (±10.29)	2.84 (±4.17)
Serum CA 19-9[a]	13.68 (±13.79)	469.51 (±1,917.72)	78.18 (±179.90)	884.56 (±2,705.64)

Abbreviations: MUC=Mucin
a – Serum CA 19-9 data was only available for 87 cases [19 Low Risk & 68 High Risk (35 High Risk IPMN and 33 Invasive IPMN/PDAC) on final pathology].

**Table S4. Post-hoc Dunn's test for differences in mucin expression and serum CA 19-9 between individual levels of the three-tiered risk classification.**			
**Marker**	**Low v. High p-Value**[a]	**High v. Invasive p-Value**[a]	**Low v. Invasive p-Value**[a]
MUC6	**0.01**	0.47	**0.004**
Serum CA 19-9[b]	**0.04**	**0.046**	**0.0003**

a – p-Values adjusted for multiple comparisons using the Benjamini-Hochberg method.
b – Serum CA 19-9 data was only available for 87 cases [19 Low Risk & 68 High Risk (35 High Risk IPMN and 33 Invasive IPMN/PDAC) on final pathology].

Table S5. Mean percent positivity of mucin expression and mean serum CA 19-9 levels according to clinicodemographic covariates.					
**Clinicodemographic Covariates**	**MUC1**	**MUC2**	**MUC5AC**	**MUC6**	**CA 19-9[a]**
**Age at Diagnosis**					
<71.7 Years	23.12 (±16.17)	6.36 (±13.89)	24.18 (±21.65)	6.35 (±9.36)	114.11 (±402.49)
≥71.7 Years	23.55 (±19.37)	8.88 (±20.16)	22.66 (±19.93)	3.82 (±5.46)	651.70 (±2,311.83)
**Sex**					
Female	20.86 (±16.68)	8.24 (±18.20)	24.49 (±21.38)	4.88 (±5.82)	594.22 (±2,319.34)
Male	25.65 (±18.56)	7.04 (±16.51)	22.41 (±20.23)	5.27 (±9.22)	212.40 (±819.37)
**Body Mass Index[b]**					
Normal Weight	24.69 (±20.66)	4.95 (±14.92)	25.27 (±22.58)	6.16 (±9.85)	506.54 (±1,556.42)
Overweight/Obese	22.43 (±16.12)	9.17 (±18.45)	22.63 (±19.78)	4.56 (±6.33)	287.16 (±1,763.92)
**Pancreatitis History**					
Yes	21.87 (±18.05)	10.38 (±20.04)	24.59 (±19.43)	4.96 (±6.97)	503.90 (±2,354.21)
No	24.27 (±17.64)	5.86 (±15.17)	22.67 (±21.62)	5.16 (±8.22)	326.31 (±1,187.77)
**New Onset Diabetes**					
Yes	17.77 (±17.36)	8.48 (±20.71)	19.26 (±21.36)	1.98 (±3.34)	560.72 (±1,861.15)
No	24.15 (±17.76)	7.50 (±16.84)	24.03 (±20.68)	5.54 (±8.09)	560.72 (±1,861.15)
**High Risk Stigmata[c]**					
Present (≥1)	29.72 (±20.47)	9.43 (±19.93)	28.29 (±22.56)	4.21 (±9.51)	683.00 (±2,418.32)
Absent	21.65 (±16.11)	7.40 (±17.46)	22.33 (±19.77)	5.70 (±6.09)	90.95 (±420.61)
**Worrisome Features[c]**					
Present (≥1)	25.99 (±19.17)	8.80 (±19.67)	26.67 (±22.04)	4.93 (±8.26)	444.15 (±1,865.89)
Absent	21.85 (±15.22)	6.04 (±12.16)	17.21 (±14.63)	5.69 (±5.47)	13.84 (±11.27)
**Duct Involvement**					
Main	25.73 (±19.19)	13.33 (±23.00)	29.64 (±23.76)	4.91 (±9.13)	462.11 (±2,062.93)
Side-Branch	21.29 (±16.33)	2.75 (±7.45)	18.12 (±16.12)	5.23 (±6.37)	323.03 (±1,271.24
**Epithelial Subtype**					
Gastric	14.76 (±11.38)	1.28 (±1.81)	16.42 (±12.03)	7.34 (±5.65)	38.74 (±82.69)
Intestinal	21.22 (±18.13)	22.41 (±27.99)	28.64 (±25.13)	4.95 (±7.97)	53.59 (±91.92)
Pancreatobiliary	25.85 (±18.56)	2.92 (±8.67)	21.01 (±16.93)	4.13 (±5.23)	584.68 (±2,268.89)
Gastric & Intestinal	23.01 (±11.19)	3.54 (±8.77)	13.84 (±14.36)	3.25 (±4.69)	16.60 (±13.05)
Gastric & Pancreatobiliary	35.01 (±19.71)	5.00 (±11.01)	36.71 (±26.64)	7.96 (±17.90)	1,220.29 (±2,445.45)
Intestinal & Pancreatobiliary	14.32 (±8.09)	12.75 (±22.39)	32.09 (±29.06)	4.35 (±4.69)	23.90 (±37.27)
Gastric, Intestinal, & Pancreatobiliary	19.34 (±18.92)	0.32 (±0.28)	14.72 (±18.84)	4.57 (±6.61)	248.73 (±413.35)

a – Serum CA 19-9 data was only available for 105 cases.
b – 1 patient with underweight BMI was excluded from this analysis.
c – 25 patients were unable to be assessed for high-risk stigmata or worrisome features due to missing data and were excluded from this analysis.

**Table S6. Optimism corrected AUCs for HRS or WFs in patients without HRS with and without MUC6 expression or its radiomic signature.**		
**Risk Model**	**Optimism Corrected AUC**	
	**Overall (N=101)**	**Patients with Radiomic Features Available (N=82)**
HRS	0.77	0.79
HRS + MUC6	0.86	0.86
HRS + MUC6 Radiomics	--	0.89
**Risk Model**	**Patients without HRS (N=56)**	**Patients without HRS and with Radiomic Features Available (N=39)**
WFs	0.70	0.71
WFs + MUC6	0.78	0.78
WFs + MUC6 Radiomics	--	0.84
Abbreviations: HRS=High-Risk Stigmata, WFs=Worrisome Features, AUC=Area Under the Curve, NR=Not Reportable<br>a – Not Reportable values indicate a failure in bootstrap internal validation due to a singular Hessian matrix.		

**Table S7. Optimism corrected AUCs for HRS or WFs in patients without HRS with and without MUC6 expression or its radiomic signature in patients with BD-IPMNs.**		
**Risk Model**	**Optimism Corrected AUC**	
	**BD-IPMNs (N=47)**	**BD-IPMN with Radiomic Features Available (N=33)**
HRS	0.66	0.66
HRS + MUC6	0.75	0.74
HRS + MUC6 Radiomics	--	0.80
**Risk Model**	**BD-IPMNs without HRS (N=37)**	**BD-IPMNs without HRS with Radiomic Features Available (N=25)**
WFs	0.70	0.70
WFs + MUC6	0.74	0.74
WFs + MUC6 Radiomics	--	0.79
Abbreviations: HRS=High-Risk Stigmata, WFs=Worrisome Features, AUC=Area Under the Curve		

**Table S8. Performance of HRS with and without tumoral MUC6 expression and its radiomic signature to discern high-risk IPMNs overall and in BD-IPMN patients.**							
	**Cohort Outcome Prevalence**				**20% Outcome Prevalence**		
**Risk Model**	**Sensitivity**	**Specificity**	**PPV**	**NPV**	**PPV**	**NPV**	**Accuracy[a]**
**BD-IPMNs (N=47)**							
HRS	0.36 (0.18-0.57)	0.95 (0.77-1.00)	0.90 (0.55-1.00)	0.57 (0.39-0.73)	0.55 (0.25-0.89)	0.85 (0.81-0.90)	65.5%
HRS + MUC6	0.68 (0.46-0.85)	0.77 (0.55-0.92)	0.77 (0.55-0.92)	0.68 (0.46-0.85)	0.54 (0.33-0.80)	0.91 (0.86-0.95)	72.5%
**BD-IPMNs with Radiomic Features Available (N=33)**							
HRS	0.39 (0.17-0.64)	0.93 (0.68-1.00)	0.88 (0.47-1.00)	0.56 (0.35-0.76)	0.50 (0.20-0.88)	0.85 (0.80-0.90)	66.0%
HRS + MUC6	0.72 (0.47-0.90)	0.73 (0.45-0.92)	0.76 (0.50-0.93)	0.69 (0.41-0.89)	0.38 (0.22-0.63)	0.90 (0.82-0.96)	72.5%
HRS + MUC6 Radiomics	0.72 (0.47-0.90)	0.87 (0.60-0.98)	0.87 (0.60-0.98)	0.72 (0.47-0.90)	0.52 (0.30-0.83)	0.92 (0.86-0.96)	79.5%

Abbreviations: HRS=High-Risk Stigmata, PPV=Positive Predictive Value, NPV=Negative Predictive Value; IDI=Integrated Discrimination Improvement
a – Accuracy represents the mean of the sensitivity and specificity.

**Table S9. Performance of WFs in the absence of HRS with and without tumoral MUC6 expression and its radiomic signature to discern high-risk IPMNs overall and in BD-IPMN patients.**							
	**Cohort Outcome Prevalence**				**20% Outcome Prevalence**		
**Risk Model**	**Sensitivity**	**Specificity**	**PPV**	**NPV**	**PPV**	**NPV**	**Accuracy[a]**
**BD-IPMNs without HRS Present (N=37)**							
WFs	0.88 (0.62-0.98)	0.52 (0.30-0.74)	0.58 (0.37-0.78)	0.85 (0.55-0.98)	0.31 (0.21-0.43)	0.93 (0.82-0.98)	70.0%
WFs + MUC6	0.94 (0.70-1.00)	0.48 (0.26-0.70)	0.58 (0.37-0.77)	0.91 (0.59-1.00)	0.31 (0.23-0.41)	0.95 (0.85-0.99)	71.0%
**BD-IPMNs with Radiomic Features Available and without HRS Present (N=25)**							
WFs	0.91 (0.59-1.00)	0.50 (0.23-0.77)	0.59 (0.33-0.82)	0.88 (0.47-1.00)	0.31 (0.20-0.45)	0.93 (0.80-0.99)	70.5%
WFs + MUC6	0.91 (0.59-1.00)	0.43 (0.18-0.71)	0.56 (0.31-0.78)	0.86 (0.42-1.00)	0.28 (0.19-0.41)	0.92 (0.79-0.99)	67.0%
WFs + MUC6 Radiomics	0.55 (0.23-0.83)	1.00 (0.77-1.00)	1.00 (0.54-1.00)	0.74 (0.49-0.91)	0.73 (0.36-0.99)	0.89 (0.84-0.95)	77.5%

Abbreviations: HRS=High-Risk Stigmata, WFs=Worrisome Features, PPV=Positive Predictive Value, NPV=Negative Predictive Value; IDI=Integrated Discrimination Improvement
a – Accuracy represents the mean of the sensitivity and specificity.

Table S10. Spearman correlation coefficients of the MUC6 and risk classification radiomic signatures.

Analytic Subset	Rho	p-Value
HRS + Radiomics Overall (N=82)	-0.52	<0.0001
WFs + Radiomics Overall (N=39)	-0.31	0.06

Table S11. Odds ratios and 95% confidence intervals for the associations of HRS, WFs in patients without HRS, a previously developed tumor risk classification radiomic signature, and the MUC6 radiomic signatures assessing for independence of risk classification and MUC6 signatures when combined with HRS or WFs in patients without HRS.		
**Model**	**Odds Ratio (95% CI)**	**p-Value**
**Radiomics Subset (N=82)**		
**HRS Present**	21.48 (3.63-127.05)	**0.0007**
**Low MUC6 Radiomics**	21.48 (3.63-127.05)	**0.0007**
**High Risk Tumor Radiomics**	6.14 (1.95-19.35)	**0.002**
**MUC6 Radiomics + Tumor Radiomics**		
Low MUC6 Radiomics	12.69 (2.43-66.25)	**0.003**
High Risk Tumor Radiomics	3.80 (1.08-13.38)	**0.04**
**HRS + MUC6 Radiomics + Tumor Radiomics**		
HRS Present	22.66 (3.24-158.68)	**0.002**
Low MUC6 Radiomics	20.86 (2.99-145.73)	**0.002**
High Risk Tumor Radiomics	5.24 (1.15-23.88)	**0.03**
**Patients without HRS Present Radiomics Subset (N=39)**		
**WFs Present**	7.72 (1.64-36.35)	**0.01**
**Low MUC6 Radiomics**	16.95 (2.56-112.46)	**0.003**
**High Risk Tumor Radiomics**	2.70 (0.85-8.62)	0.09
**MUC6 Radiomics + Tumor Radiomics**		
Low MUC6 Radiomics	20.90 (2.88-151.72)	**0.003**
High Risk Tumor Radiomics	5.06 (1.02-25.08)	**0.047**
**WFs + MUC6 Radiomics + Tumor Radiomics**		
WFs Present	5.12 (0.81-32.36)	0.08
Low MUC6 Radiomics	14.76 (2.29-94.99)	**0.005**
High Risk Tumor Radiomics	2.83 (0.56-14.29)	0.21
Abbreviations: CI=Confidence Interval; HRS=High Risk Stigmata, WFs=Worrisome Features		
