# Supplementary material for: CT-Derived Radiomic Signature of MUC6 Expression Improves Guideline-Based Risk Stratification in Intraductal Papillary Mucinous Neoplasms
Source: Cancers (Basel). 2026 Jul 15;18(14):2264. doi: 10.3390/cancers18142264 (PMC13406335; doi:10.3390/cancers18142264)
Supplement: Supplementary file 1 [file cancers-18-02264-s001.zip › Supplementary Materials/Supplemental Figures.pdf]

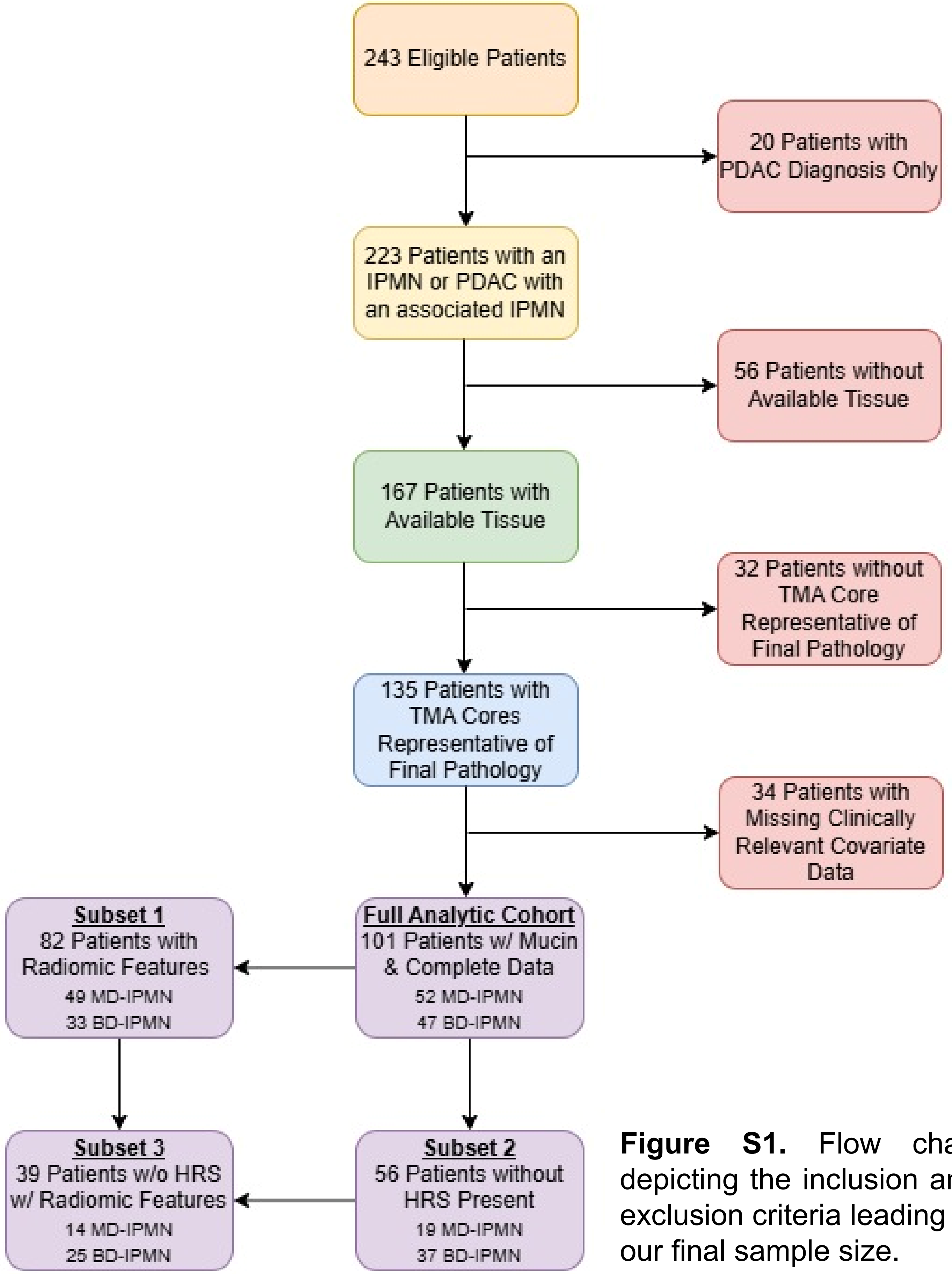

Figure S1. Flow chart depicting the inclusion and exclusion criteria leading to our final sample size.

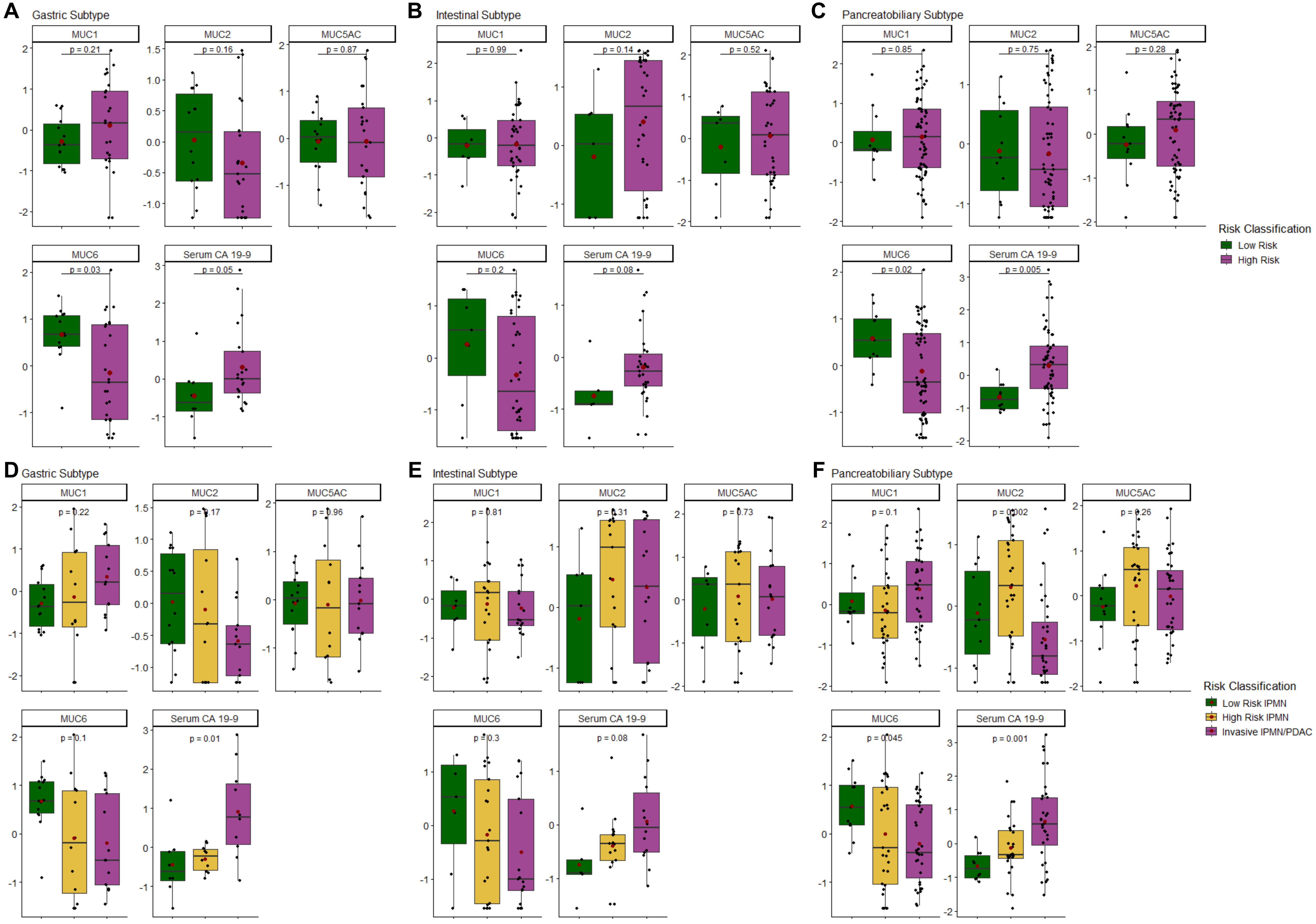

Figure S2. Boxplots showing Box-Cox transformed and z-score standardized mean percent positivity of mucin expression and serum CA 19-9 according to dichotomous (A-C) and three-level pathologic risk classification (D-F) in subgroups of patients with a gastric (A/D), intestinal (B/E), or pancreatobiliary (C/F) subtype.

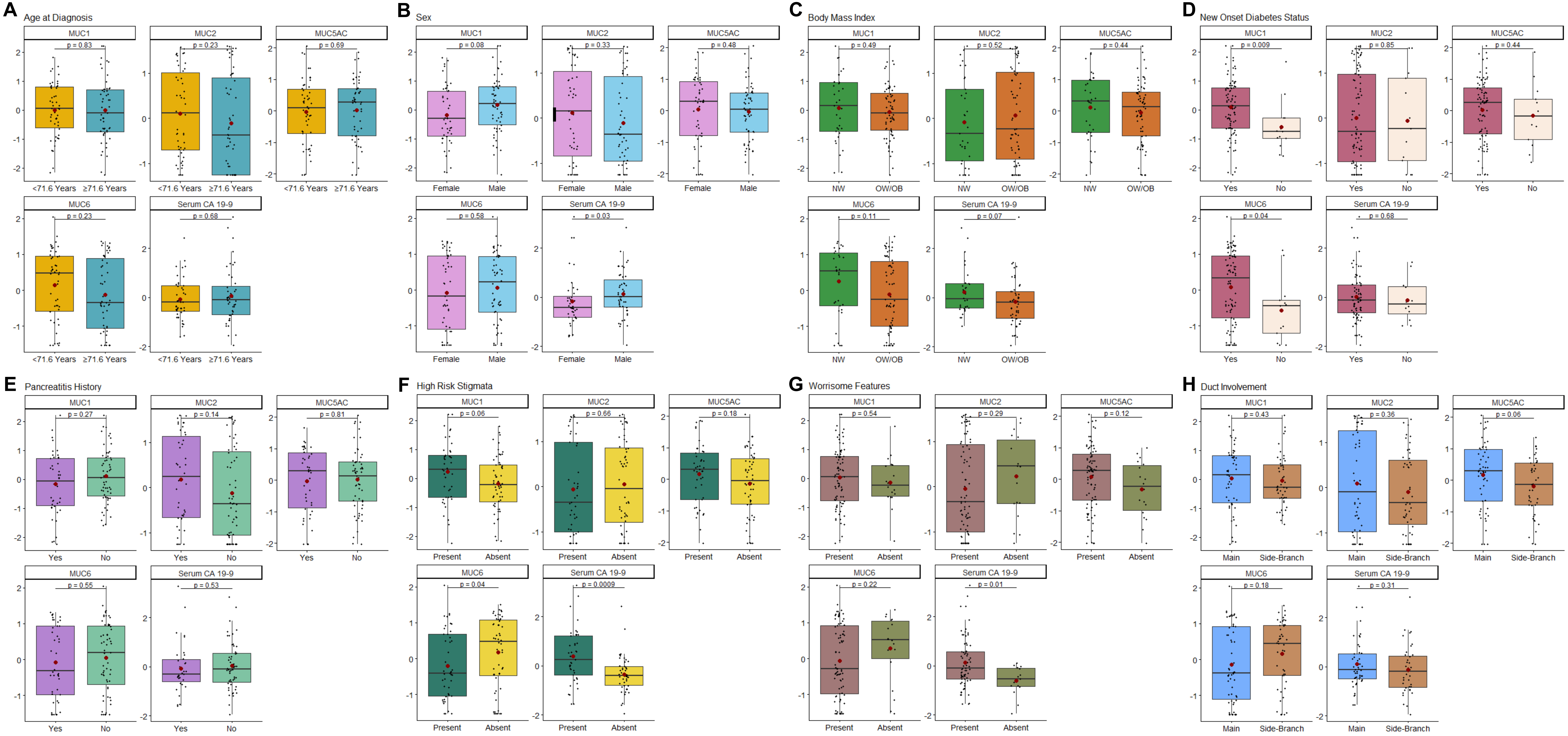

Figure S3. Box plots showing mean percent positivity of mucin and other biomarker expression in pancreatic tumors according to several clinicodemographic factors including A) age at diagnosis, B) sex, C) body mass index, D) new onset diabetes, E) history of pancreatitis, F) high-risk stigmata, G) worrisome features, and H) duct involvement.

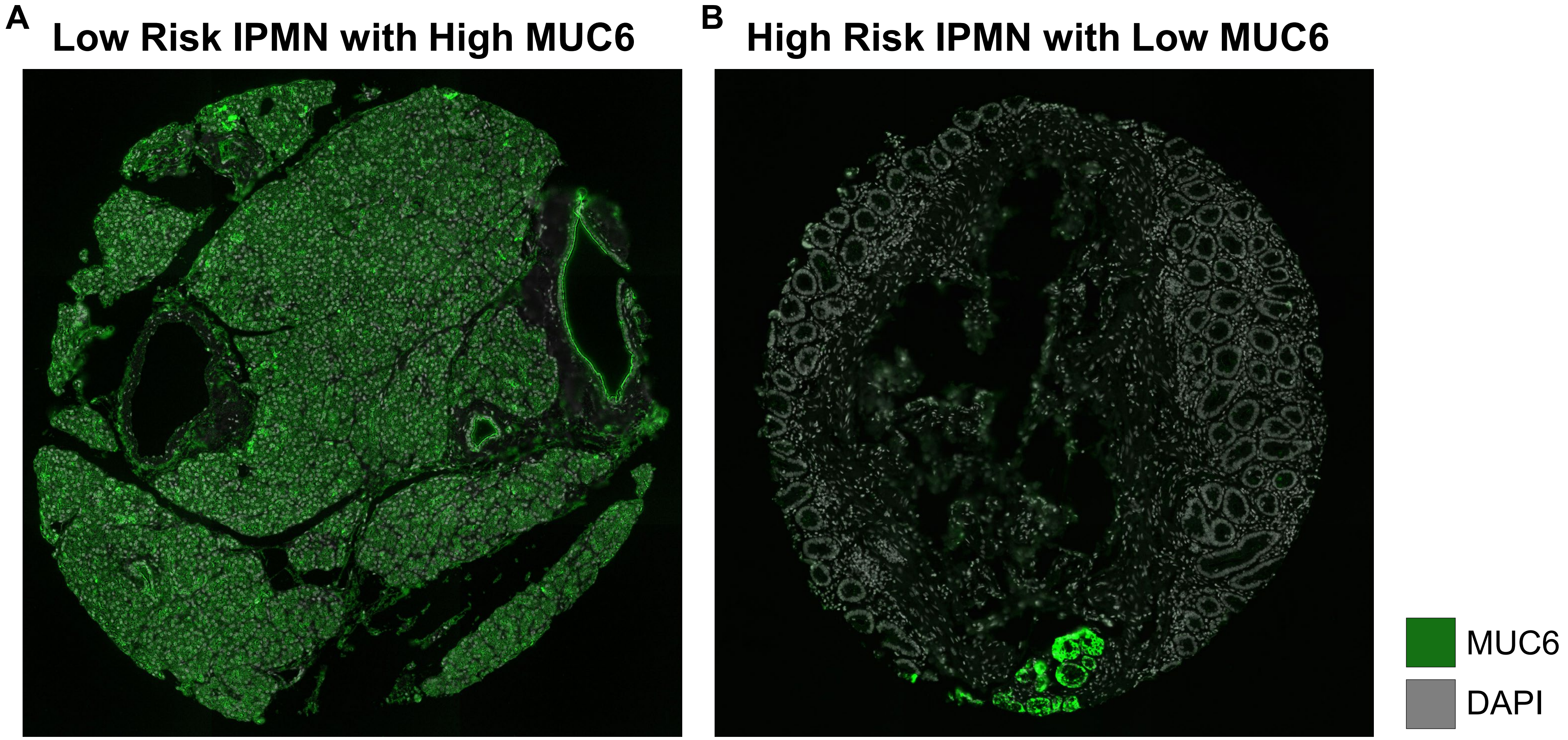

Figure S4. Tissue core images reflective of our central finding of higher MUC6 expression in patients with A) low risk lesions and lower expression in patients with B) high-risk lesions using the 1.53% MUC6 positivity threshold.

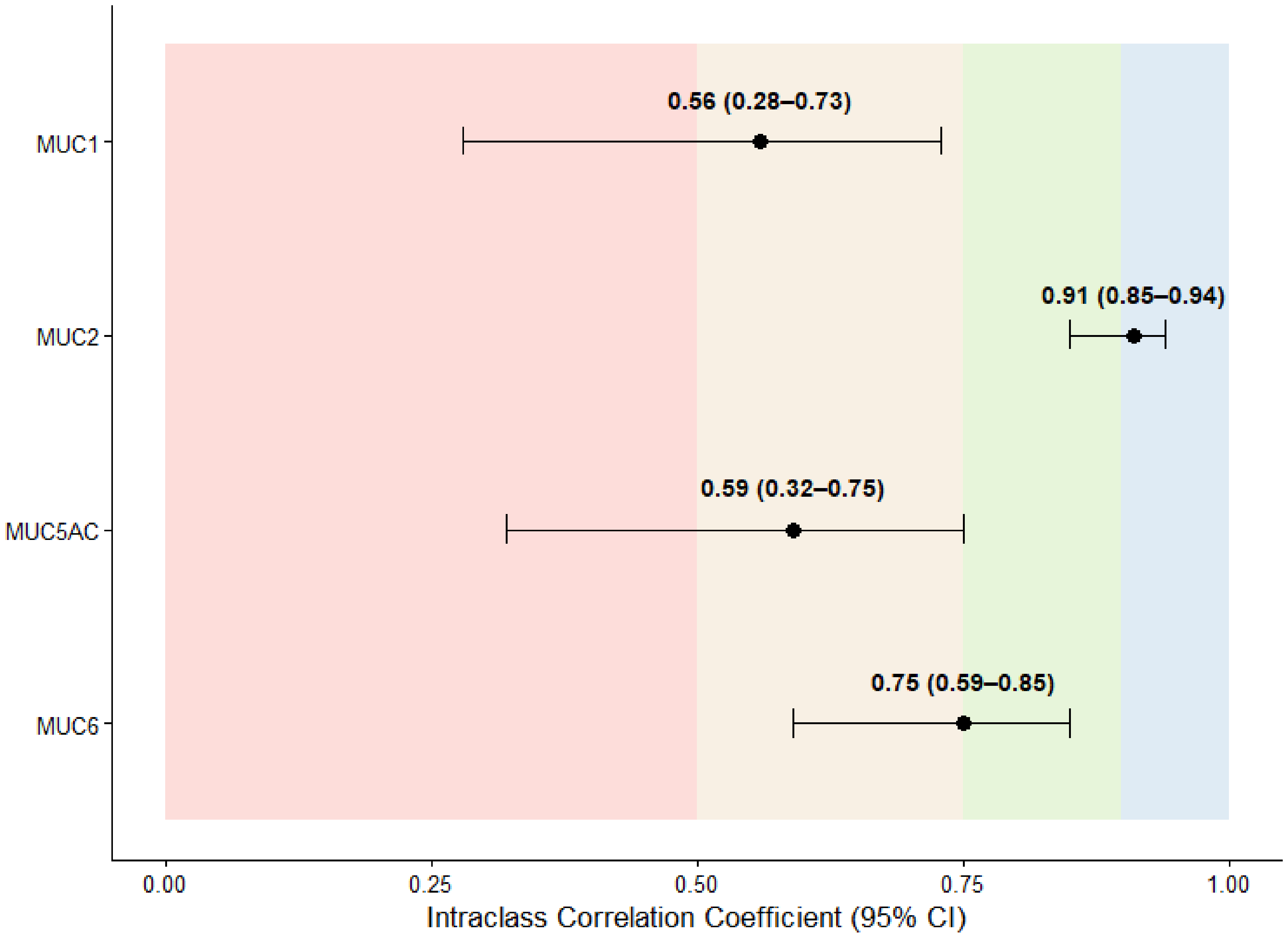

Figure S5. Variability in mucin expression per tissue core is presented as a forest plot of the intraclass correlation coefficient and their 95% confidence intervals (CIs). Variability between duplicate cores is minimal for MUC2 and MUC6, but high for MUC1 and MUC5AC.

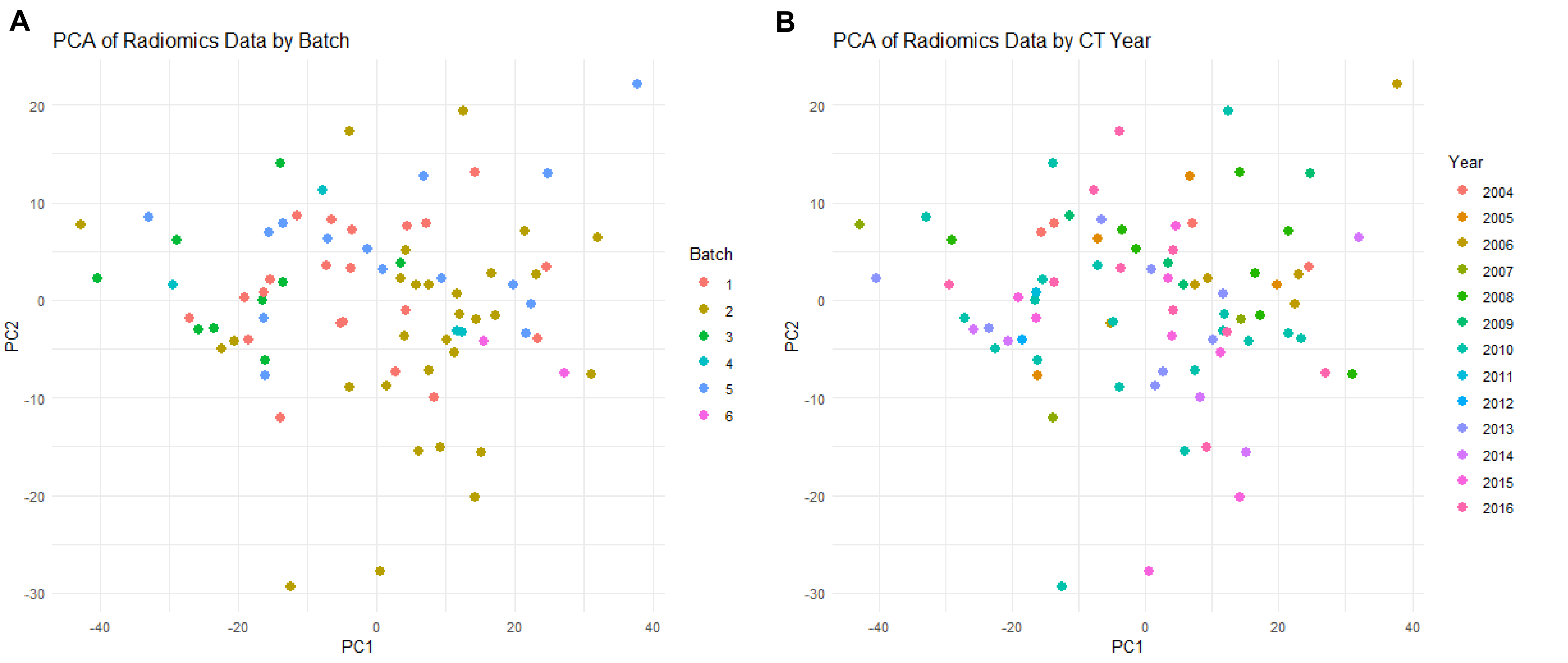

Figure S6. Analysis of batch effects by A) analytic batch and B) year of computed tomography (CT) scan effects across all radiomic features. Principal components analysis (PCA) scatterplot reveals no clustering by radiomics batch nor by year the CT scan was performed.

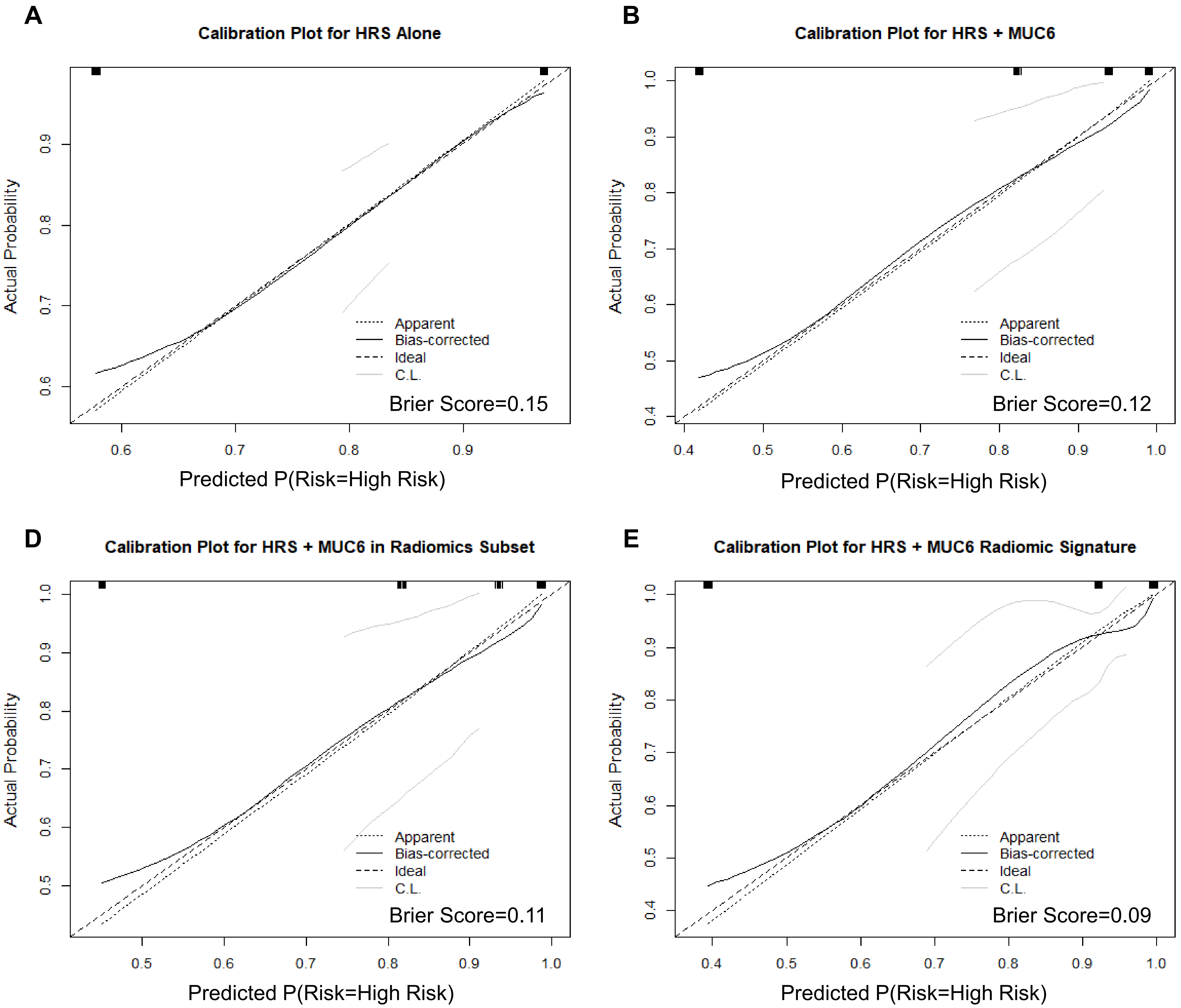

Figure S7. Calibration plots and Brier scores of penalized logistic regression models of A) HRS alone and B) HRS with tumoral MUC6 expression in the overall study population along with C) HRS alone, D) HRS with tumoral MUC6 expression, and E) HRS with the MUC6 radiomic signature in the subset with radiomics data.

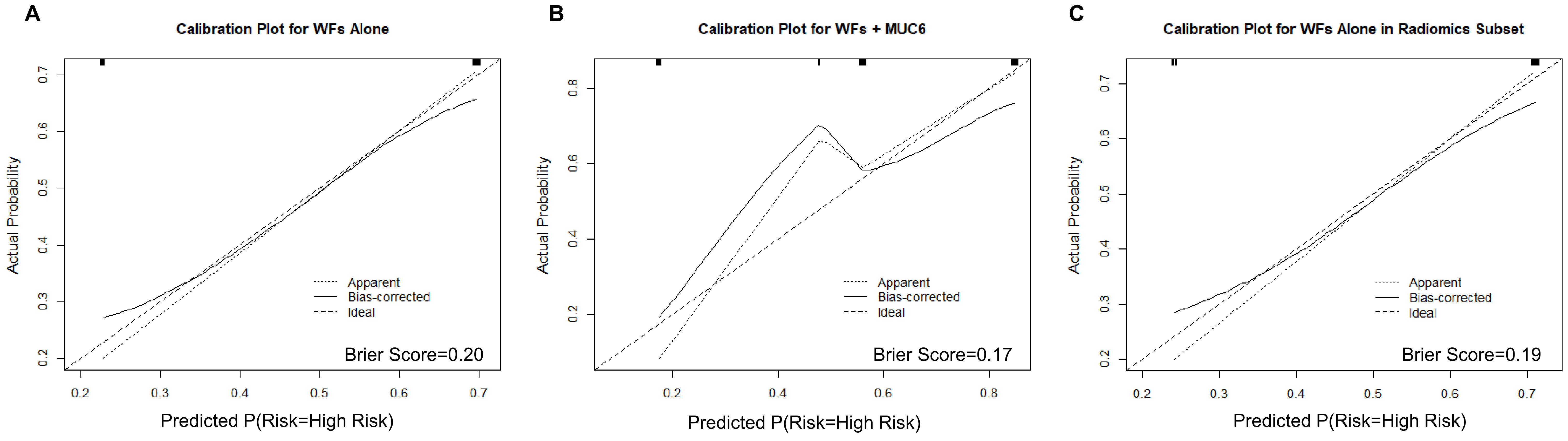

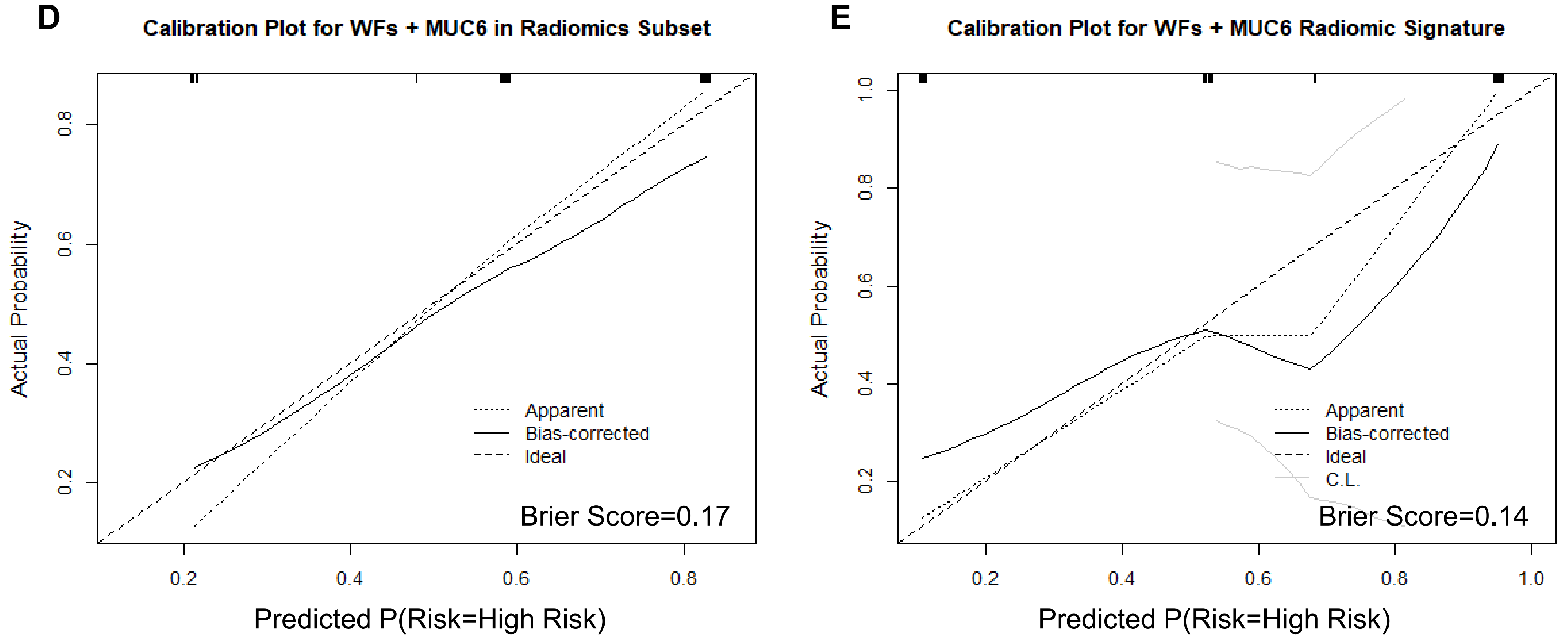

Figure S8. Calibration plots and Brier scores of penalized logistic regression models of A) WFs alone and B) WFs with tumoral MUC6 expression in the subset of patients without HRS present along with C) WFs alone, D) WFs with tumoral MUC6 expression, and E) WFs with the MUC6 radiomic signature in the subset of patients without HRS present and radiomics data.

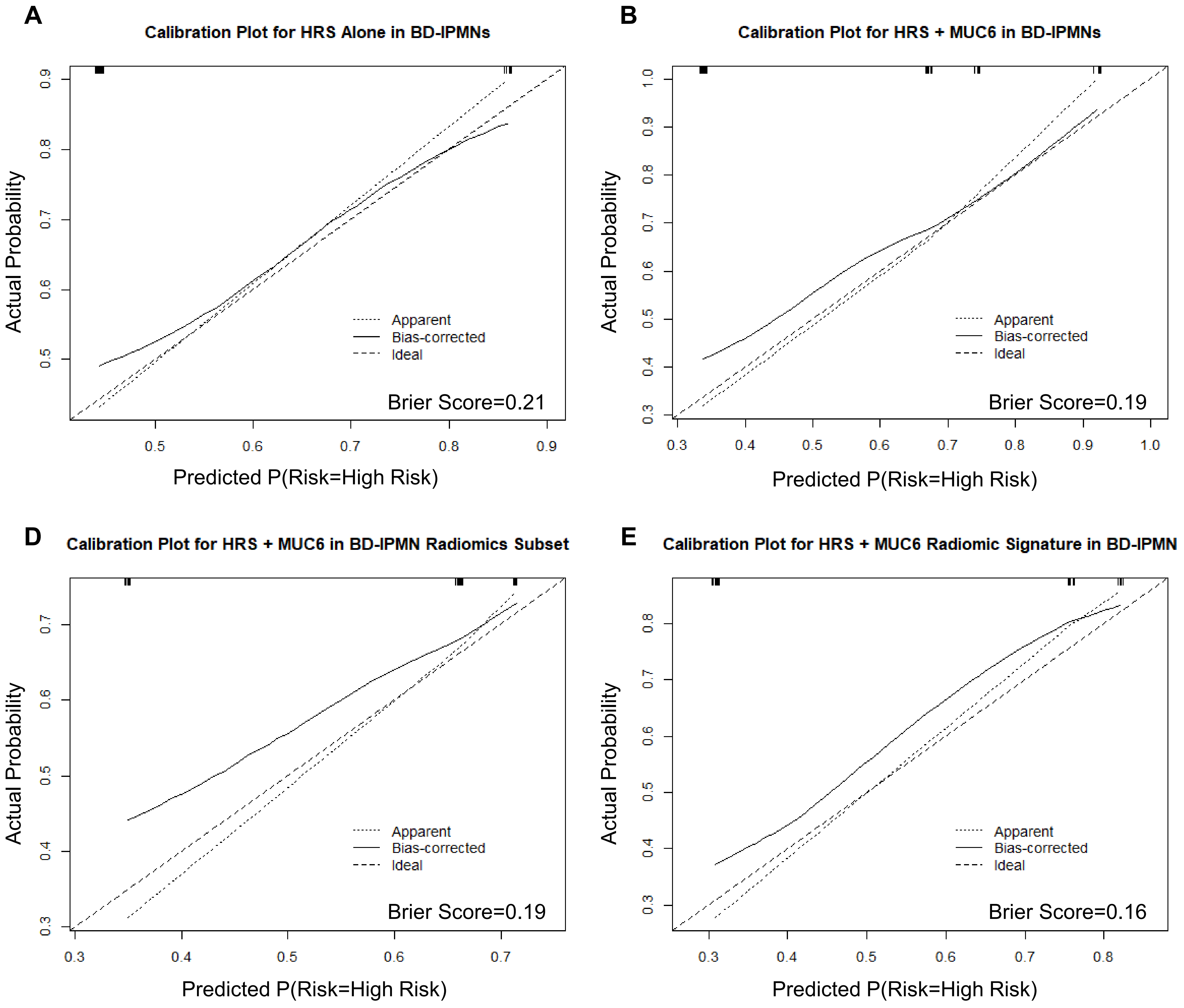

Figure S9. Calibration plots and Brier scores of penalized logistic regression models of A) HRS alone and B) HRS with tumoral MUC6 expression in patients with BD-IPMNs along with C) HRS alone, D) HRS with tumoral MUC6 expression, and E) HRS with the MUC6 radiomic signature in the BD-IPMN subset with radiomics data.

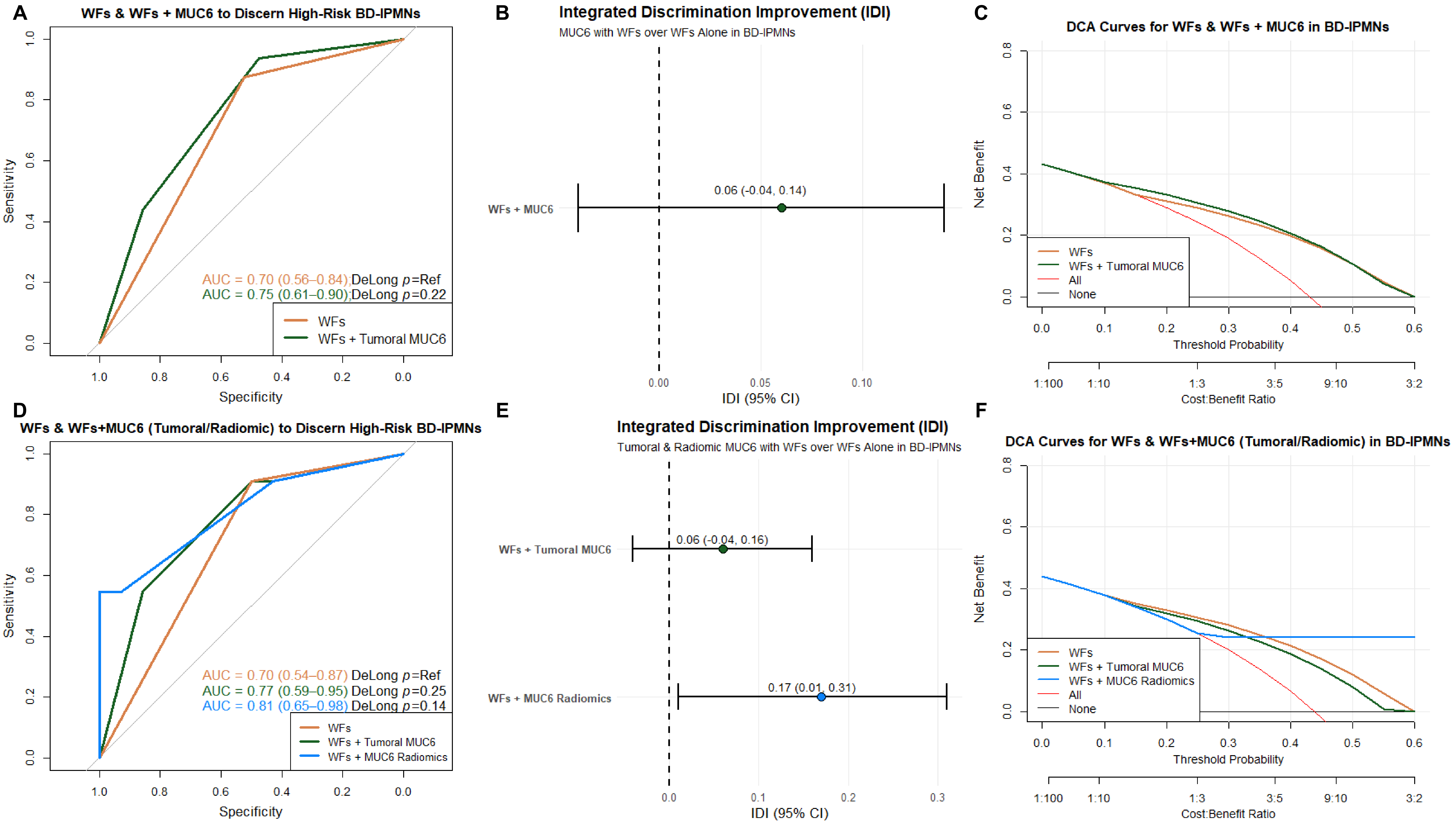

Figure S10. The utility of including tumoral MUC6 expression (A-C) and its radiomic signature (D-F) with guideline-based WFs in patients without HRS present for identifying BD-IPMN patients with high-risk pathology. ROC curves (A &D) depict the discrimination of WFs with and without MUC6 expression or its radiomic signature while forest plots (B & E) quantify the discrimination improvement for including MUC6 expression or its radiomic signature over WFs alone. Finally, DCA curves (C & F) demonstrate the clinical utility of including MUC6 expression or its radiomic signature with WFs to aid in treatment decisions.

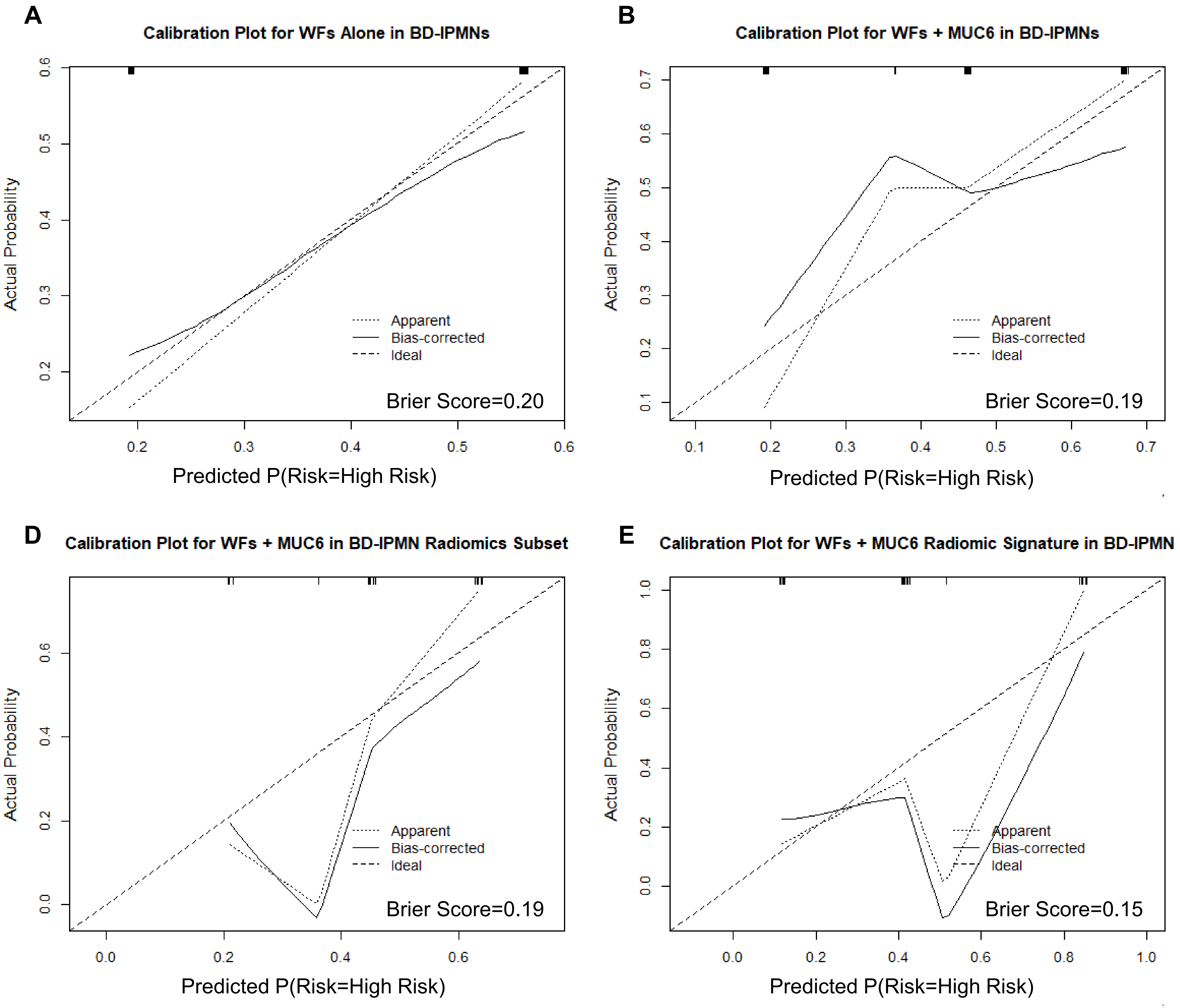

Figure S11. Calibration plots and Brier scores of penalized logistic regression models of A) WFs alone and B) WFs with tumoral MUC6 expression in the subset of BD-IPMN patients without HRS present along with C) WFs alone, D) WFs with tumoral MUC6 expression, and E) WFs with the MUC6 radiomic signature in the subset of BD-IPMN patients without HRS present and radiomics data.
