# Supplementary material for: CT-Derived Radiomic Signature of MUC6 Expression Improves Guideline-Based Risk Stratification in Intraductal Papillary Mucinous Neoplasms
Source: Cancers (Basel). 2026 Jul 15;18(14):2264. doi: 10.3390/cancers18142264 (PMC13406335; doi:10.3390/cancers18142264)
Supplement: Supplementary file 1 [file cancers-18-02264-s001.zip › Supplementary Materials/Supplemental Methods.pdf]

Supplemental Methods

Multiplex Immunofluorescence. Sections were baked at 65°C for three hours then transferred to the BOND RX. All subsequent steps (e.g., deparaffinization, antigen retrieval) were performed using an automated OPAL IHC procedure (AKOYA). OPAL staining of each antigen occurred as follows: heat induced epitope retrieval (HIER) was achieved with HIER-EDTA pH 9.0 buffer for twenty minutes at 95°C before the slides were blocked with AKOYA blocking buffer for ten minutes. Then slides were incubated with primary antibody, MUC1 [Cell Marque, MRQ-17 (RRID:AB_1160625), HIER-EDTA pH 9.0, 1:400, dye 570] at room temperature for one hour followed by OPAL HRP polymer and one of the OPAL fluorophores during the final TSA step. Individual antibody complexes were stripped after each round of antigen detection. This was repeated three more times using the following antibodies: MUC2 [Cell Marque, MRQ-18 (RRID:AB_1160642), HIER- EDTA pH 9.0, 1:800, dye520], MUC5AC [Cell Marque, MRQ-19 (RRID:AB_1160649), HIER-EDTA pH 9.0, 1:1,200, dye 650], and MUC6 [Cell Marque, MRQ-20 (RRID:AB_1160667), HIER-EDTA pH 9.0, 1:600, dye620]. After the final stripping step, DAPI counterstain was applied to the multiplexed slide and removed from BOND RX for cover slipping with ProLong Diamond Antifade Mountant (ThermoFisher Scientific).

Antibody Validation. During the Opal Multiplex-IHC panel design, commercially available monoclonal antibodies suitable for FFPE-IHC and with high epitope sensitivity were selected. The initial antibody validation and titration was performed in control tissue (tonsil) and IPMN tissue by Diaminobenzidine (DAB)-IHC. Isotope control was performed in control and IPMN tissue samples using the same staining parameters to

determine the contribution of non-specific background to staining. Opal Multiplex-IHC protocols is designed to reduce the impact of epitope instability and “spectral bleeding”. Opal fluorophore was applied to each marker while avoiding locating spectrally adjacent fluorophore with the same cell and/or compartment to reduce spectral artifacts. Next, epitope stability was assessed by Opal monoplex-IHC at different protocol position while avoiding successive antibodies colocalized in the same cell and/or compartment to reduced incomplete stripping interference. An extra-stripping step was introduced to the standard Opal Multiplex-IHC protocols to eliminate issues with incomplete stripping. A final antibody and TSA titration using Opal monoplex-IHC control and RCC samples was used to achieve signal intensity in desired rage to eliminate spectral bleeding during image unmixing and analysis. Finally, the Opal Multiplex-IHC panel was validated in control tissue and IPMN tissue samples with an additional drop-control and Isotype-Multiplex-IHC control to ensure no TSA inhibition and non-specific background.
